# Mechanisms for Robust Local Differential Privacy

**DOI:** 10.3390/e26030233

**Published:** 2024-03-06

**Authors:** Milan Lopuhaä-Zwakenberg, Jasper Goseling

**Affiliations:** Faculty of Electrical Engineering, Mathematics and Computer Science, University of Twente, 7522 NB Enschede, The Netherlands

**Keywords:** local differential privacy, Rényi divergence, robust optimization

## Abstract

We consider privacy mechanisms for releasing data X=(S,U), where *S* is sensitive and *U* is non-sensitive. We introduce the robust local differential privacy (RLDP) framework, which provides strong privacy guarantees, while preserving utility. This is achieved by providing robust privacy: our mechanisms do not only provide privacy with respect to a publicly available estimate of the unknown true distribution, but also with respect to similar distributions. Such robustness mitigates the potential privacy leaks that might arise from the difference between the true distribution and the estimated one. At the same time, we mitigate the utility penalties that come with ordinary differential privacy, which involves making worst-case assumptions and dealing with extreme cases. We achieve robustness in privacy by constructing an uncertainty set based on a Rényi divergence. By analyzing the structure of this set and approximating it with a polytope, we can use robust optimization to find mechanisms with high utility. However, this relies on vertex enumeration and becomes computationally inaccessible for large input spaces. Therefore, we also introduce two low-complexity algorithms that build on existing LDP mechanisms. We evaluate the utility and robustness of the mechanisms using numerical experiments and demonstrate that our mechanisms provide robust privacy, while achieving a utility that is close to optimal.

## 1. Introduction

We consider the setting in which an aggregator collects data from many users with the purpose of, for instance, computing statistics or training a machine learning model. In particular, the data contain sensitive information and users do not trust the aggregator. Therefore, they employ a privacy mechanism that transforms the data before sending it to the aggregator. Users have data X=(S,U) from a finite alphabet X=S×U, where s∈S is sensitive information and u∈U is non-sensitive. Data are distributed i.i.d. across users according to the distribution $P^{*}$. In order to preserve their privacy, users disclose a sanitized version *Y* of *X* by using a privacy mechanism Q:X→Y. The aim is that *Y* contains as much information about *X* as possible without leaking too much information about *S*. The challenge that is addressed in this paper is to develop good privacy mechanisms. This scenario and closely related ones were studied in, for instance [1,2,3,4,5,6,7,8,9,10,11]. In this paper, we use the following version of local differential privacy (LDP), as introduced in [3]:

(1) $$P\left(Y=y|S=s\right)\leq e^{\varepsilon }P\left(Y=y|S=s^{\prime }\right),$$

for all $s,s^{\prime }\in S$ and privacy parameter ε>0. In addition, we measure the utility of *Y* through the mutual information I(X;Y). We discuss differences with related work in Section 2.

Note that if all information is sensitive, i.e., if X=S, (1) reduces to

(2) $$P\left(Y=y|X=x\right)\leq e^{\varepsilon }P\left(Y=y|X=x^{\prime }\right),$$

which is the traditional LDP constraint [1,2,5]. An important property of (2) is that it does not depend on $P^{*}$, but only on Q. The independence of $P^{*}$ is a key factor in the success of differential privacy, since it leverages the need to make assumptions about the distribution of the data or on the background/side-knowledge available to the aggregator. As is clear from (1), however, independence from $P^{*}$ no longer holds if not all data are sensitive.

Assuming that $P^{*}$ is known, one can develop good privacy mechanisms for various settings with partially sensitive information [3,6,12]. In practice, however, $P^{*}$ has to be modeled using domain knowledge or estimated from data, leading to errors. The prevalent approach in the literature has been to develop privacy mechanisms based on a (point) estimate $\hat{P}$ and analyze sensitivity with respect to. errors in this estimate. In this work, we follow the approach that was proposed in [13,14], which is to construct a set F of probability distributions that we are confident contains $P^{*}$. Subsequently, we construct privacy mechanisms that aim to maximize utility, while satisfying (1) for all probability distributions in F. We call the resulting privacy framework robust local differential privacy (RLDP).

In a sense, RLDP is a relaxed form of privacy. Indeed, it may seem appealing, but it is—as we illustrate next—often infeasible to enforce (1) for all possible distributions. To this end, we consider two extreme cases. First, consider a joint distribution of *S* and *U* under which S=U. Intuitively, we cannot disclose much information about *U*, since this is directly leaking information about *S*. As such, the utility of *Y* is low. Next, consider a joint distribution under which *S* and *U* are independent. Intuitively, we can disclose *U* without additional precautions, providing a high utility on *Y*. The point is that we need to design a single privacy mechanism Q that satisfies (1) for all distributions, including the ‘worst case’ in which S=U, leading to low utility *Y*. In this work, we take the mid-ground between, on the one hand, only using a point estimate $\hat{P}$ and, on the other hand, using all possible distributions. We do so by defining a set of ‘reasonable’ distributions F. In particular, we construct F based on public side-information. This public side information consists of *n* pairs of data $\left(s_{1},u_{1}\right),\ldots ,\left(s_{n},u_{n}\right)$, which like the data of users are i.i.d. according to unknown distribution $P^{*}$. Our set F is constructed as a closed ball under a Rényi divergence around the maximum likelihood point estimate $\hat{P}$ of $P^{*}$. By doing so, we are (statistically) confident that F contains $P^{*}$, with the radius of the ball controlling the confidence level.

The RLDP framework is an instance of the more general Pufferfish framework [15]. In Section 2, we make this connection explicit and use it to describe the semantic privacy guarantees that are offered by RLDP.

The main contributions of this paper are as follows:

1. We use a Rényi divergence to construct F and analyze the resulting structure and statistics of F. In particular, we demonstrate that projections of F are again balls under the same divergence. Moreover, we bound the projected sets in terms of an $l_{1}$ norm.
2. Using these results we approximate F by an enveloping polytope. We then use techniques from robust optimization [16,17,18] to characterize PolyOpt, the mechanism that is optimal over this polytope.
3. A drawback of this method is that it relies on vertex enumeration and is, therefore, computationally unfeasible for large alphabets. Therefore, we introduce two low-complexity privacy mechanisms. The first is independent reporting (IR), in which *S* and *U* are reported through separate LDP mechanisms.
4. We characterize the conditions that underlying LDP mechanisms have to satisfy in order for IR to ensure RLDP. Furthermore, while IR can incorporate any LDP mechanism, we show that it is optimal to use randomized response [19]. This drastically reduces the search space and allows us to find the optimal IR mechanism using low-dimensional optimization.
5. The second low-complexity mechanism that we develop is called secret-randomized response (SRR) and is based on randomized response.
6. We show that SRR maximizes mutual information in the low-privacy regime for the case that F is the entire probability simplex.
7. We demonstrate the improved utility of RLDP over LDP with numerical experiments. In particular, we compare the performance of our mechanisms with generalized random response [5]. We provide results for both synthetic data sets and real-world census data.

The structure of this paper is as follows: After discussing related work in Section 2, we describe the model in detail in Section 3. In Section 4, we present results on the structure and statistics of projections of F. These results are used in Section 5 to develop the PolyOpt privacy mechanism. Low-complexity privacy mechanisms are presented in Section 6 and Section 7. In Section 8, we evaluate the discussed methods experimentally. Finally, in Section 9, we provide a discussion of our results and provide an outlook on future work. Most proofs are deferred to Appendix A.

Part of this paper was presented at the IEEE International Symposium on Information Theory 2021 [14]. In this paper, we generalize from a $\chi^{2}$-divergence to an arbitrary Rényi divergence. Moreover, Section 4 and Section 6, most of Section 8, and all proofs are new in the current paper.

## 2. Related Work

### 2.1. The Pufferfish Framework

Our RLDP framework is an instance of the more general Pufferfish framework [15]. In this subsection, we make this connection explicit and elaborate on the semantic guarantees offered by RLDP.

A privacy definition following the Pufferfish framework specifies (i) a set of potential secrets, (ii) a set of discriminative pairs of secrets, and (iii) a set of assumptions about how data are generated. In RLDP the potential secrets are the possible values of *S*, i.e., S. We want to prevent the aggregator from learning anything about *S*. This means that it should not be able to distinguish the case S=s from $S=s^{\prime }$ for all $s\neq s^{\prime }$, so all non-identical pairs are discriminative. Note that this relies on S being finite, with extensions to continuous S discussed in detail in [15].

The set of assumptions on how data are generated consist, in our setting, of probability distributions over X. A key idea in Pufferfish is that this set explicitly models the information that is available to an attacker, i.e., an entity that is trying to infer information about *S* by observing *Y*. In our setting, the aggregator is the only attacker and a probability distribution *P* over X captures the beliefs that the attacker has about *S* prior to seeing *Y*. We can rewrite (1) as

(3) $$\frac{P_{X∼P}\left(S=s|Y=y\right)}{P_{X∼P}\left(S=s^{\prime }|Y=y\right)}\leq e^{\varepsilon }\frac{P_{X∼P}\left(S=s\right)}{P_{X∼P}\left(S=s^{\prime }\right)}$$

and see that our local differential privacy constraint (1) can be interpreted as the condition that the posterior distribution of *S* after seeing *Y* must be very close to the prior distribution. The relevance of *P* is that it captures a specific set of beliefs of the attacker. As such, we want (3) to hold for various values of *P*, where each *P* captures specific background/side-knowledge available to the attacker/aggregator. Note that by doing so we are not making any claims about the actual knowledge available to the aggregator, but instead describing the possible scenarios for which we want to protect the privacy of users. In Pufferfish, these possible scenarios are called the set of assumptions on how data are generated, and in RLDP this is F.

Often, side-information in the form of domain knowledge or existing data is publicly available; i.e., to both the users and the aggregator. This public side-information may suggest, for instance, that there is, at most, limited dependence between *S* and *U*. In that case, protecting against attackers who have the belief that S=U incurs an enormous penalty in achieved utility. It is true that those attackers gain a lot of information on *S* by observing *Y*. However, they could have also obtained this information from the public side-information directly. Therefore, the approach taken in the Pufferfish framework and in this paper is that we only protect against attackers that have beliefs, i.e., distributions *P*, that are in line with publicly available side information.

A challenge in working with the Pufferfish framework is that it is often challenging to find good mechanisms. A general mechanism is proposed in [20], but it relies on enumerating over all distributions in F, which is an uncountable set in our setting and cannot be used here. A constrained version of Pufferfish that facilitates analysis and a methodology for finding good mechanisms is proposed in [21]. Another interesting line of work is to model correlations between users in the non-local differential privacy setting [22]. Finally, ref. [23] proposed a modeling framework for capturing domain knowledge about the data. In contrast, in the current work, we impose constraints that are learned from data. Our setting does not fit any of the frameworks for which good mechanisms are known in the literature. One of the main contributions of this paper is to develop such mechanisms.

### 2.2. Other Privacy Frameworks

Disclosing *X* through a privacy mechanism that protects sensitive information *S* has been studied extensively. One line of work starts from differential privacy [24] and imposes the additional challenge that the aggregator cannot be trusted, leading to the concept of local differential privacy [1,2,5]. For this setting, several privacy mechanisms exist, including randomized response [19] and unary encoding [25]. Optimal LDP mechanisms under a variety of utility metrics, including mutual information, are found in [5]. In [1,2,5], all data are sensitive, i.e., X=S. The variation of LDP for the case of disclosing X=(S,U), where only *S* is sensitive, was proposed in [3] and is the setting that we study in this paper. Another line of work connects this setting to the information bottleneck [26], leading to a privacy constraint in terms of mutual information [6,8,9,10]. In these works, it is shown that approaches to optimizing the information bottleneck also work for finding good privacy mechanisms.

Next to differential privacy and mutual information as privacy measures, a multitude of other privacy frameworks and leakage measures exist [27]. Some of these have been studied in the context of privacy mechanisms. In [7,11], privacy leakage is measured through the improved potential of statistical inference by an attacker after seeing the disclosed information. This measure is formulated through a general cost function, with mutual information resulting as a special case. Perfect privacy, which demands the output to be independent of the sensitive data, was studied in [28], and methods were given to find optimal mechanisms in this setting. An estimation-theoretic framework was studied in [29,30]. Our use of a Rényi divergence in the construction of F may suggest considering a generalization of our privacy definition. This could be achieved by considering, for instance, a Rényi divergence in the privacy constraint, as done in [31]. Along a different line, in [32], the maximal leakage measure with a clear operational interpretation is defined. In [33], this measure is generalized to a parametrized measure, enabling interpolating between maximal leakage and mutual information. A stronger, pointwise, version of the maximal leakage measure is proposed in [34]. These are interesting research directions but not pursued in this paper.

Our setting X=(S,U) is a special case of a Markov chain S−X−Y, where only *X* is observed. This Markov chain is typically studied in the information bottleneck and privacy funnel settings [6,26]. We do not generalize to this setting, because we need observations of *S* for the estimate of $P_{U|S}$. Without direct observations of *s*, we can only make worst-case assumptions on $P_{U|S}$, leading to very poor utility. A different type of model, in which only part of the information in *X* is sensitive, is proposed in [12]. This is a block-structured model in which *X* is partitioned and information about the partition of an element is sensitive but its index in the partition is not. Our setting of X=S×U does not fit this model. One can partition X according to U, but our privacy constraints are different from [12]. We will elaborate on this in Section 6.

### 2.3. Robustness

The distribution $P_{S,U}^{*}$ is not available in practice. The approach taken in most works is to estimate $P_{S,U}^{*}$ from data and analyze sensitivity with respect to this estimate ${\hat{P}}_{S,U}$. One of the contributions in [7] is to quantify the impact of mismatched priors, i.e., the impact of not knowing $P_{S,U}^{*}$ exactly. A bound on the resulting level of privacy is derived in terms of the total variational distance between the actual and the estimated ${\hat{P}}_{S,U}$. The setting in [35] is similar to ours: A ball of probability distributions, centered around a point estimate, was defined that contains $P_{S,U}^{*}$ with high probability. It was then shown that a privacy mechanism that was designed based on the empirical distribution was valid for the entire set for a looser privacy constraint. The privacy slack was quantified and shown to approach zero as the size of the data set increased. An important difference with the current work was that we explicitly optimize the privacy mechanism over the uncertainty set. Another difference is that we base our ball on a Rényi divergence, whereas [35] used an $l_{1}$ norm. The main technical tool used in [35] was large deviations theory, whereas we rely on convex analysis and robust optimization. We also mention [36,37]. In [36] it is assumed that nothing is known about $P_{S}^{*}$ and $P_{U|S}^{*}$. It is shown that good privacy mechanisms can be found through a connection to maximal correlation, see also [38]. In [37], sets of probability distributions are not derived from data but carefully modeled such that optimal mechanisms can be derived analytically.

Using robust optimization [16] to find a good mechanism that satisfies privacy constraints for all $P_{S,U}$ in uncertainty set F was proposed in [13,14]. In this work, we generalize and extend results from [14]. The idea of robust optimization is that constraints in an optimization problem contain uncertain parameters that are known to come from a (a priori defined) uncertainty set. The constraints must hold for possible values of the uncertain parameters. A key result is that, using Fenchel duality, the problem can be expressed in terms of the support function of the uncertainty set and the convex conjugate of the constraint [16,17]. The case where the uncertain parameters are probabilities is known as distributionally robust optimization. Using results from [39], it was shown in [40] how an uncertainty set can be constructed from data using an *f*-divergence, providing an approximate confidence set. Confidence sets for parameters that are not necessarily probabilities were constructed in [18] under a $\chi^{2}$-divergence. Convergence of robust optimization based on *f*-divergences was studied in [41] and for the case of a KL-divergence in [42]. In [43], it is shown how distributionally robust optimization problems over Wasserstein balls can be reformulated as convex problems. For the regular differential privacy setting, distributionally robust optimization was used in [44] to find optimal additive privacy mechanisms for a general perturbation cost function. In this paper, we show how robust optimization can be applied to the setting of partially sensitive information with local differential privacy.

### 2.4. Miscellaneous

Another line of work on privacy mechanisms builds on recent advances in generative adversarial networks [45]. In [46,47], a generative adversarial framework is used to provide privacy mechanisms that do not use explicit expressions for $P_{X}$. Even though this is not explicitly addressed in [46,47], it is expected that the generalization properties of networks will provide a form of robustness. Closely related approaches are used in the field of face recognition [48,49], with the aim of preventing biometric profiling [50]. The leakage measures that are used in [48,49], however, do not seem to have an operational interpretation.

Disclosing information in a privacy-preserving way is one of the main challenges in official statistics [51,52]. The setting considered in the current paper is closely connected to disclosing a table with microdata, where each record in the table is released independently of the other records. This approach to disclosing microdata was studied in [4] by considering expected error as the utility measure and mutual information as the privacy measure. The resulting optimization problem corresponds to the traditional rate-distortion problem.

## 3. Model and Preliminaries

In this section, we give an overview of the setting and objectives of this paper. The notation used in this section, as well as the rest of the paper, is summarized in Table 1.

The data space is X=S×U, where S and U are finite sets. We write $\left|S\right|=:a_{1}$, $\left|U\right|=:a_{2}$, and $\left|X\right|=a_{1}a_{2}=:a$. Data items X=(S,U) are drawn from a probability distribution $P^{*}$ in $P_{X}$, the space of probability distributions on X; here, *S* represents sensitive data, while *U* represents non-sensitive data. The aggregator’s aim is to create a privacy mechanism Q:X→Y such that Y=Q(X) contains as much information about *X* as possible, while not leaking too much information about *S*.

The mechanism Q is a probabilistic map, which we represent by a left stochastic matrix ${\left(Q_{y|x}\right)}_{y\in Y,x\in X}$, and we write |Y|=b. Often, we identify Y={1,…,b}, and likewise for other sets.

The distribution $P^{*}$ is not known exactly. Instead, there is a set of possible distributions $F\subset P_{X}$, where $P_{X}$ denotes the probability simplex over X. We choose F in such a way that it is likely that $P^{*}\in F$. The uncertainty set F captures our uncertainty about $P^{*}$, we guarantee privacy for all P∈F. We denote this as robust local differential privacy (RLDP).

**Definition 1**  (Robust Local Differential Privacy)**.** *Let ε≥0 and $F\subset P_{X}$. We say that Q satisfies (ε,F)-RLDP if for all $s,s^{\prime }\in S$, all y∈Y, and all P∈F we have*

(4) $$P_{X∼P}\left(Y=y|S=s\right)\leq e^{\varepsilon }P_{X∼P}\left(Y=y|S=s^{\prime }\right).$$

Note that we use the notation $P_{X∼P}\left(•\right)$ to emphasize that *X* is distributed according to *P*. If no confusion can arise, we often leave out the subscript X∼P, to improve readability. Note that we can also write

(5) $$P_{X∼P}\left(Y=y|S=s\right)=\sum_{u\in U}Q_{y|s,u}P_{X∼P}\left(U=u|S=s\right),$$

so Definition 1 depends on the conditional probabilities of *U* given S=s and $S=s^{\prime }$. It does not, however, depend on the realization of *U*.

For clarity and use in future sections, we give the definition of regular LDP [1], which is used when the goal is to obfuscate all of *X*, rather than just *S*.

**Definition 2**  (Local Differential Privacy)**.** *Let ε≥0. We say that Q:X→Y satisfies ε-LDP if for all $x,x^{\prime }\in X$ and all y∈Y we have*

(6) $$P\left(Y=y|X=x\right)\leq e^{\varepsilon }P\left(Y=y|X=x^{\prime }\right).$$

Now, for aggregator uncertainty about $P^{*}$, as captured by F, we suppose there is a data base $\vec{x}=\left(x_{1},\cdots ,x_{n}\right)$ accessible to the user, where each $x_{I}=\left(s_{I},u_{I}\right)$ is drawn independently from $P^{*}$. Based on this, the user produces an estimate $\hat{P}$ of $P^{*}$. In the experiments, we consider a maximum likelihood estimator, i.e., ${\hat{P}}_{x}=\left|\left\{i\leq n:x_{I}=x\right\}\right|$. We construct the uncertainty set F as a closed ball around $\hat{P}$. In particular, let $D_{\alpha }$ be the Rényi divergence of order α on $P_{X}$, i.e., for α∈(0,∞)

(7) $$D_{\alpha }(\hat{P}\left||P\right)=\left\{\begin{array}{cc} \frac{1}{\alpha -1}\log\left(\sum_{x\in X}\frac{{\hat{P}}_{x}^{\alpha }}{P_{x}^{\alpha -1}}\right), & \;\mathrm{if}\;\alpha \neq 1,\\ \sum_{x\in X}{\hat{P}}_{x}\log\frac{{\hat{P}}_{x}}{P_{x}}, & \;\mathrm{if}\;\alpha =1. \end{array}\right.$$

The case α=1 follows, in fact, as a limit from the α≠1 case. Similarly, the definition can be extended to α∈{0,∞} by taking the corresponding limits, but in this paper we restrict our attention to α∈(0,∞) to keep the presentation clear. Note that $D_{1}=D_{\mathrm{KL}}$, the Kullback–Leibler divergence, and $D_{2}=\log\chi^{2}$, where the $\chi^{2}$-divergence is $\chi^{2}(P_{1}\left|\right|P_{2})=\sum_{x}{\left(P_{1,x}-P_{2,x}\right)}^{2}P_{2,x}^{-1}$. In general, a Rényi divergence is a continuous increasing function of a power divergence (a.k.a. Hellinger divergence) [39,53,54], an example of an *f*-divergence. We omit α from the notation when it is clear from the context.

We define F by fixing a bound B∈[0,∞] and letting

(8) $$F=\left\{P\in P_{X}:D_{\alpha }(\hat{P}\left||P\right)\leq B\right\}.$$

Since a Rényi divergence is a continuous increasing function of an *f*-divergence, it follows from [39,40] that F is a confidence set for $P^{*}$. In particular, for the case of α=2, which will be used in our numerical experiments in Section 8, for suitable *B*, we have

(9) $$F=\left\{P\in P_{X}:\sum_{x}\frac{{\left({\hat{P}}_{x}-P_{x}\right)}^{2}}{P_{x}}\leq \frac{F_{\chi^{2},a-1}^{-1}\left(1-\beta \right)}{n}\right\},$$

with β∈(0,1), where $F_{\chi^{2},a-1}$ is the cumulative density function of the $\chi^{2}$-distribution with a−1 degrees of freedom, resulting in a set F with significance level β. This means that the probability of $P^{*}\in F$ is at least 1−β.

Hence, by designing Q based on F, we are confident in satisfying (1) for all attackers that have beliefs that are based on the public side-information, as well as for attackers that have beliefs that are closer to $P^{*}$.

As a special case of the above, we will study the case that nothing is known about $P^{*}$. In this case, B→∞ and $F=P_{X}$. Regarding privacy, this is the ‘safest’ choice, as we do not make assumptions about $P^{*}$. Another special case is where F is a singleton, which reflects a situation where B=0 and $P^{*}$ is assumed to be known. This setting was studied in [3].

**Figure 1:**
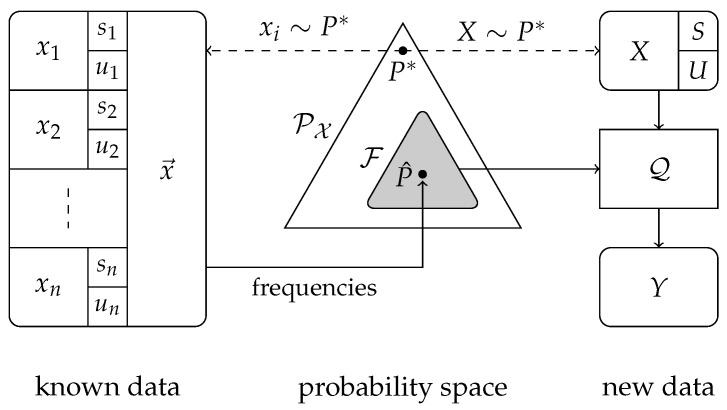
An overview of the setting of this paper when F is a confidence set based on a data set $\vec{x}$. Note that it is typically, but not necessarily, true that $P^{*}\in F$.

Given F and ε, the goal is now to create a Q:X→Y to be used on new/future data; our setting is depicted in Figure 1. The aim of this paper is to find a satisfactory answer to the following problem:

**Problem 1.**  *Given F and ε, find a Q satisfying (ε,F)-RLDP, while maximizing a given utility function.*

Throughout this paper, we follow the original privacy funnel [6] and its LDP counterpart [3] in taking mutual information I(X;Y) as a utility measure. As is argued in [6], mutual information arises naturally when minimizing log loss distortion in the privacy funnel scenario. As a utility measure of Q, we take $I_{X∼P}\left(X;Y\right)$ (abbreviated to $I_{P}\left(X;Y\right)$), since the aim is to create *Y* that reflects *X* as faithfully as possible. This utility measure depends on the distribution *P* of *X* that we choose to evaluate. Ideally, one would like to use $P=P^{*}$, but in practice this is not possible, as $P^{*}$ is unknown. In the theoretical part of this paper, we circumvent this issue by proving our results for general *P*. In the experiments of Section 8, we take $P=\hat{P}$ as the best available alternative to $P=P^{*}$. We investigate the effect of this choice by comparing $I_{P^{*}}\left(X;Y\right)$ to $I_{\hat{P}}\left(X;Y\right)$.

Another option is to use the robust utility measure $\min_{P\in F}I_{P}\left(X;Y\right)$ to ensure good utility for every ‘reasonable’ *P*, see [13]. We do not explicitly study this measure in this paper, but since our results hold for general *P*, they can also be applied to robust utility.

**Example 1.**  *We set up an example to illustrate the concepts of this paper. Take $S=\{s_{1},s_{2}\}$ and $U=\{u_{1},u_{2}\}$, and suppose*

(10) $$P^{*}=\left(\begin{array}{c} P_{s_{1},u_{1}}^{*}\\ P_{s_{1},u_{2}}^{*}\\ P_{s_{2},u_{1}}^{*}\\ P_{s_{2},u_{2}}^{*} \end{array}\right)=\left(\begin{array}{c} 0.1\\ 0.1\\ 0.2\\ 0.6 \end{array}\right).$$

*Moreover, suppose we have a publicly known database of n=100 entries, from which we estimate*

(11) $$\hat{P}=\left(\begin{array}{c} {\hat{P}}_{s_{1},u_{1}}\\ {\hat{P}}_{s_{1},u_{2}}\\ {\hat{P}}_{s_{2},u_{1}}\\ {\hat{P}}_{s_{2},u_{2}} \end{array}\right)=\left(\begin{array}{c} 0.07\\ 0.10\\ 0.26\\ 0.57 \end{array}\right).$$

*To obtain a 95%-confidence set for F according to a $\chi^{2}$-distribution, we take α=2 and $B=\log\left(1+\frac{F_{\chi^{2},3}^{-1}\left(0.05\right)}{100}\right)=0.0752$. In this way, we obtain*

(12)F=P∈PX:Dα(P^||P)≤B(13)=P∈PX:log∑xP^x2Px≤log1+Fχ2,3−1(0.05)100(14)=P∈PX:∑x(P^x−Px)2Px≤Fχ2,3−1(0.95)100,

which is the desired confidence set (note that the $\chi^{2}$-distribution has |X|−1=3 degrees of freedom). In this case, we have $D_{2}(\hat{P}\left|\right|P^{*})=0.0281<B$, so $P^{*}\in F$.

## 4. Conditional Projection of F

In Section 5 and Section 7 below, we will introduce privacy mechanisms that provide (ε,F)-RLDP. These mechanisms depend on the conditional projections of F on $P_{U}$ given S=s, denoted as $F_{U|s}$. In this section, we analyze the structure and statistics of these sets. To do so, we introduce, for s∈S, u∈U and $P\in P_{X}$.

(15)Ps=∑u∈UPu,s,(16)Pu|s=Pu,sPs,(17)PU|s=(Pu|s)u∈U∈PU,(18)FU|s={PU|s:P∈F}⊂PU,

We are interested in the following statistics:

(19)Lu|s(F)=minR∈FU|sRuforagivenu∈U,(20)rads(F)=maxR∈FU|s||R−P^U|s||1.

In (19), $R_{u}$ is the *u*-coefficient of $R\in P_{U}$. It turns out that these statistics give us the information required to construct (ε,F)-protocols efficiently: In Section 5, we use $L_{u|s}\left(F\right)$ to approximate $F_{U|s}$ by a polytope, to make computation easier, while in Section 7, we use ${\mathrm{rad}}_{s}\left(F\right)$ as a measure for the size of $F_{U|s}$. While these statistics (or bounds for them) are relatively easy to find for F itself, the hard part lies in the fact that we have to give bounds for the projection $F_{U|s}$. The extent to which these bounds can be found explicitly heavily depends on the divergence measure that is used to construct F. In this section, we show how these bounds can be obtained for our case where we construct F using a Rényi divergence. The reason for this, as we will see below, is that we can give an explicit description of $F_{U|s}$.

### 4.1. Structure of $F_{U|s}$

Recall that, for a given α∈(0,∞), the Rényi divergence $D_{\alpha }:P_{X}\to \left[0,\infty \right)$ is defined by

(21) $$D_{\alpha }(\hat{P}\left||P\right)=\left\{\begin{array}{cc} \frac{1}{\alpha -1}\log\left(\sum_{x\in X}\frac{{\hat{P}}_{x}^{\alpha }}{P_{x}^{\alpha -1}}\right), & \;\mathrm{if}\;\alpha \neq 1,\\ \sum_{x\in X}{\hat{P}}_{x}\log\frac{{\hat{P}}_{x}}{P_{x}}, & \;\mathrm{if}\;\alpha =1. \end{array}\right.$$

The following theorem states that the conditional projections of balls defined by Rényi divergence are themselves Rényi divergence balls:

**Theorem 1.**  *Let s∈S be such that ${\hat{P}}_{s}>0$. Let F be defined by Rényi divergence, i.e.,*

(22) $$F=\left\{P\in P_{X}:D_{\alpha }(\hat{P}\left||P\right)\leq B\right\}$$

*for a given α∈(0,∞) and $B\in R_{\geq 0}$. Define the constant $B_{s}$ by*

(23) $$B_{s}=\left\{\begin{array}{cc} \frac{\alpha }{\alpha -1}\log\left(\frac{e^{(\alpha -1)B/\alpha }-\left(1-{\hat{P}}_{s}\right)}{{\hat{P}}_{s}}\right), & \;\mathrm{if}\;\alpha \neq 1,\\ \frac{B}{{\hat{P}}_{s}}, & \;\mathrm{if}\;\alpha =1. \end{array}\right.$$

*Then,*

(24) $$F_{U|s}=\left\{R\in P_{U}:D_{\alpha }({\hat{P}}_{U|s}\left||R\right)\leq B_{s}\right\}.$$

This theorem gives us a direct description of the $F_{U|s}$, which is useful because the $L_{u|s}\left(F\right)$ of (19) and ${\mathrm{rad}}_{s}\left(F\right)$ of (20) are defined in terms of these projection sets. A similar bound could also be found for the limit cases α=0,∞, but this is not pursued in this paper, because it does not provide additional insights.

A key property of the Rényi divergence that allows us to prove Theorem 1 is that we can write

(25) $$\frac{{\hat{P}}_{x}^{\alpha }}{P_{x}^{\alpha -1}}=\frac{{\hat{P}}_{u|s}^{\alpha }}{P_{u|s}^{\alpha -1}}\cdot \frac{{\hat{P}}_{s}^{\alpha }}{P_{s}^{\alpha -1}}.$$

This allows us to express the divergence $D_{\alpha }({\hat{P}}_{U|s}\left|\right|P_{U|s})$ in terms of $D_{\alpha }(\hat{P}\left||P\right)$. For other divergences, which may depend on $\hat{P}$ and *P* in a more complicated way, this is typically not possible. Therefore, we cannot generalize our results to uncertainty sets constructed from, for instance, arbitrary *f*-divergences.

In light of this theorem and the fact that in the following sections we care more about the statistics of $F_{U|s}$ than about those of F itself, one might be inclined to think that it is more straightforward to estimate the ${\hat{P}}_{U|s}$ from the data and defining uncertainty sets $F_{U|s}$ around them directly, without going through the intermediate stage F. However, projecting these sets back to $P_{X}$ results in a larger set. In other words, there are distributions *P* such that each $P_{U|s}$ is an element of $F_{U|s}$, while P∉F. That is, we have $F⊊F^{\prime }:=\left\{P\in P_{X}:\forall s\;P_{U|s}\in F_{U|s}\right\}$. The reason for this is that, in the proof of Theorem 1, it becomes clear that the P∈F that project to the boundary points of $F_{U|s}$ satisfy $P_{U|s^{\prime }}={\hat{P}}_{U|s^{\prime }}$ for $s^{\prime }\neq s$. In other words, elements of F can be extremal in, at most, one $F_{U|s}$. By contrast, $F^{\prime }$ also includes *P* that are extremal in multiple $F_{U|s}$. We conclude that constructing the $F_{U|s}$ directly results in a larger $F^{\prime }$, which results in a lower utility. We will give an example of this phenomenon in Example 2.

### 4.2. Statistics of $F_{U|s}$

In this section, we analyze statistics of $F_{U|s}$. More concretely, to find $L_{u|s}\left(F\right)$ and ${\mathrm{rad}}_{s}\left(F\right)$, fix *s*, α and *B* and define for ρ∈[0,1] and $\xi \in R_{\geq 0}$ such that ξ(1−ρ)≤1,

(26)φBs(ρ,ξ)=1α−1logρξ1−α+(1−ρ)1−ρξ1−ρ1−α−Bs,if α≠1andρ≠1,ρlog1ξ+(1−ρ)log1−ρ1−ρξ−Bs,if α=1andρ≠1,log1ξ−Bs,if ρ=1,(27)ξ−(ρ)=infξ∈(0,1]:φBs(ρ,ξ)≤0,(28)ξ+(ρ)=supξ∈[1,(1−ρ)−1):φBs(ρ,ξ)≤0.

Note that the case ρ=1 can be obtained via taking the limit. The expressions for $\xi_{-}$ and $\xi_{+}$ are a bit complicated, but note that, given ρ<1, the function $\varphi_{B_{s}}\left(\rho ,\xi \right)$ is convex in ξ. Thus, $\varphi_{B_{s}}\left(\rho ,\xi \right)=0$ has at most two solutions. Furthermore, $\varphi_{B_{s}}\left(\rho ,1\right)=-B_{s}$ and $\varphi_{B_{s}}\left(\rho ,\xi \right)\to \infty$ as ξ approaches 0 or $\frac{1}{1-\rho }$, so for ρ<1 the values $\xi_{-}\left(\rho \right)$ and $\xi_{+}\left(\rho \right)$ are the two solutions to $\varphi_{B_{s}}\left(\rho ,\xi \right)=0$.

The following proposition expresses our desired statistics in terms of $\xi_{-}$ and $\xi_{+}$.

**Proposition 1.**  *Let u∈U. Then,*

(29)Lu|s(F)=P^u|sξ−(P^u|s),(30)rads(F)=2maxU1⊂U:U1≠⌀P^U1|s(ξ+(P^U1|s)−1).

As discussed above, $\xi_{\pm }\left(\rho \right)$ can be found quickly numerically; however, the calculation of ${\mathrm{rad}}_{s}\left(F\right)$ still involves taking the maximum over an exponentially large set.

### 4.3. Special Case α=2

In this section, we show that when α=2, we can find explicit expressions for $\xi_{\pm }$ and consequently $L_{u|s}$ and ${\mathrm{rad}}_{s}$. As discussed in (9), for this α, the set F is a confidence set for a $\chi^{2}$-test. To find $\xi_{-}\left(\varrho \right),\xi_{+}\left(\varrho \right)$, we need to solve $\varphi_{B_{s}}\left(\rho ,\xi \right)=0$. For α=2, we can write this as a quadratic equation in ξ, and solving it leads to the following expression:

**Lemma 1.**  *Suppose α=2. Then,*

(31) $$\begin{array}{cc} \xi_{-}\left(\rho \right) & =\frac{e^{B_{s}}+2\rho -1-\sqrt{\left(e^{B_{s}}-1\right)\left(e^{B_{s}}-{\left(2\rho -1\right)}^{2}\right)}}{2e^{B_{s}}\rho }, \end{array}$$

(32) $$\begin{array}{cc} \xi_{+}\left(\rho \right) & =\frac{e^{B_{s}}+2\rho -1+\sqrt{\left(e^{B_{s}}-1\right)\left(e^{B_{s}}-{\left(2\rho -1\right)}^{2}\right)}}{2e^{B_{s}}\rho }. \end{array}$$

Now, we can determine $L_{u|s}\left(F\right)$ and ${\mathrm{rad}}_{s}\left(F\right)$ using Lemma 1 and Proposition 1. For $L_{u|s}\left(F\right)$, we immediately obtain an expression; for ${\mathrm{rad}}_{s}\left(F\right)$, a careful analysis of $\xi_{+}$ shows that the optimal $U_{1}$ of (30) can be found. For large enough $B_{s}$, the optimum is at $U_{1}=\left\{u_{\min}\right\}$, where $u_{\min}$ is the *u* that minimizes ${\hat{P}}_{u|s}$. Thus, we obtain a concrete expression for ${\mathrm{rad}}_{s}\left(F\right)$ without the need for optimization. For smaller $B_{s}$, we do not find an exact expression, but we can still derive a lower bound. The results are summarized in the following proposition.

**Proposition 2.**  *Let α=2. Then, the following hold:*

*1.*  *One has*
(33) $$L_{u|s}\left(F\right)=\frac{e^{B_{s}}+2{\hat{P}}_{u|s}-1-\sqrt{\left(e^{B_{s}}-1\right)\left(e^{B_{s}}-{\left(2{\hat{P}}_{u|s}-1\right)}^{2}\right)}}{2e^{B_{s}}}.$$
*2.*  *Let $u_{\min}=\arg\min_{u\in U}{\hat{P}}_{u|s}$. If $B_{s}\geq \log\left(1+{\left(1-{\hat{P}}_{u_{\min}|s}\right)}^{2}\right)$, then*
(34) $${\mathrm{rad}}_{s}\left(F\right)=\frac{-e^{B_{s}}+2{\hat{P}}_{u_{\min}|s}-1+\sqrt{\left(e^{B_{s}}-1\right)\left(e^{B_{s}}-{\left(2{\hat{P}}_{u_{\min}|s}-1\right)}^{2}\right)}}{e^{B_{s}}}.$$
*3.*  *If $B_{s}<\log\left(1+{\left(1-{\hat{P}}_{u_{\min}|s}\right)}^{2}\right)$, one has ${\mathrm{rad}}_{s}\left(F\right)\leq \sqrt{e^{B_{s}}-1}$.*

We note that α=2 is not the only value of α for which one can bound $L_{u|s}$ and ${\mathrm{rad}}_{s}$. For instance, for α≤1, one can use Pinsker’s inequality [55,56] and its generalizations [57] to bound ${\mathrm{rad}}_{s}\left(F\right)$ in terms of $\left|\right|{\hat{P}}_{U|s}-P_{U|s}{\left|\right|}_{1}$, which in turn can be used to bound $L_{u|s}\left(F\right)$. However, unlike α=2, these do not result in exact bounds.

**Example 2.**  *We continue Example 1. We have*

$$\begin{array}{cccccc} {\hat{P}}_{s_{1}} & =0.17, & {\hat{P}}_{u_{1}|s_{1}} & =0.4118, & {\hat{P}}_{u_{2}|s_{1}} & =0.5882,\\ {\hat{P}}_{s_{2}} & =0.83, & {\hat{P}}_{u_{1}|s_{2}} & =0.3133, & {\hat{P}}_{u_{2}|s_{2}} & =0.6867. \end{array}$$

*Inserting our values of B and ${\hat{P}}_{s}$ into Theorem 2, we find $B_{s_{1}}=0.3782$, $B_{s_{2}}=0.0900$. In other words,*

(35) PU|s1=R=Ru1Ru2∈PU:D20.41180.5882||Ru1Ru2≤0.3782,

(36) PU|s2=R=Ru1Ru2∈PU:D20.31330.6867||Ru1Ru2≤0.0900.

*To determine the lower bounds on each $R_{u_{i}}$, we use Proposition 2 to obtain*

$$\begin{array}{cccc} L_{u_{1}|s_{1}}\left(F\right) & =0.1620, & L_{u_{2}|s_{1}}\left(F\right) & =0.2829,\\ L_{u_{1}|s_{2}}\left(F\right) & =0.1923, & L_{u_{2}|s_{2}}\left(F\right) & =0.5337. \end{array}$$

*In principle, we can also use Proposition 2 to determine the ${\mathrm{rad}}_{s}\left(F\right)$. However, in this case, there is a more straightforward approach. Since |U|=2, every element of $F_{U|s}$ is a vector of length two whose coefficients sum to 1; thus $P_{U|s}$ is determined by $P_{u_{1}|s}$. Since $L_{u_{1}|s}\left(F\right)\leq P_{u_{1}|s}\leq 1-L_{u_{2}|s}\left(F\right)$, it follows that*

$$\begin{array}{cc} F_{U|s_{1}} & ≅[L_{u_{1}|s_{1}}\left(F\right),1-L_{u_{2}|s_{1}}\left(F\right)]=\left[0.1620,0.7171\right],\\ F_{U|s_{2}} & ≅[L_{u_{1}|s_{2}}\left(F\right),1-L_{u_{2}|s_{2}}\left(F\right)]=\left[0.1923,0.4663\right]. \end{array}$$

*Under this identification, ${\mathrm{rad}}_{s}\left(F\right)$ is only twice the maximal distance from ${\hat{P}}_{u_{1}|s}$ to the endpoint of this interval (the factor two comes from the fact that $\left|\right|P_{U|s}-{\hat{P}}_{U|s}{\left|\right|}_{1}=|P_{u_{1}|s}-{\hat{P}}_{u_{1}|s}\left|+\right|P_{u_{2}|s}-{\hat{P}}_{u_{2}|s}\left|=2\right|P_{u_{1}|s}-{\hat{P}}_{u_{1}|s}|$). Hence,*

$$\begin{array}{cc} {\mathrm{rad}}_{s_{1}}\left(F\right) & =2\max\{0.4118-0.1620,0.7171-0.4118\}=0.6107,\\ {\mathrm{rad}}_{s_{1}}\left(F\right) & =2\max\{0.3133-0.1923,0.4663-0.3133\}=0.3061. \end{array}$$

*We can also construct the set $F^{\prime }=\left\{P\in P_{X}:\forall s\;P_{U|s}\in F_{U|s}\right\}$ of Section 4.1. We can write this as*

(37) $$F^{\prime }=\left\{\left(\begin{array}{c} P_{s_{1},u_{1}}\\ P_{s_{1},u_{2}}\\ P_{s_{2},u_{1}}\\ P_{s_{2},u_{2}} \end{array}\right)\in P_{X}:\begin{array}{c} 0.1620\leq P_{u_{1}|s_{1}}\leq 0.7171,\\ 0.1923\leq P_{u_{1}|s_{2}}\leq 0.4663 \end{array}\right\}.$$

*The inequality $0.1620\leq P_{u_{1}|s_{1}}$ can be written as $0.1620\leq \frac{P_{s_{1},u_{1}}}{P_{s_{1},u_{1}}+P_{s_{1},u_{2}}}$, or $0.1620P_{s_{1},u_{2}}\leq 0.83830P_{s_{1},u_{1}}$; in other words, this becomes a linear constraint. We can do the same for the other constraints and these, together with inequality constraints of the form $P_{s,u}\geq 0$ and the equality constraint $\sum_{s,u}P_{s,u}=1$, define the polytope $F^{\prime }\subset R^{4}$. One can calculate that this polytope is a simplex, spanned by the vertices*

(38) $$\left(\begin{array}{c} 0.7171\\ 0.2829\\ 0\\ 0 \end{array}\right),\left(\begin{array}{c} 0.1620\\ 0.8380\\ 0\\ 0 \end{array}\right),\left(\begin{array}{c} 0\\ 0\\ 0.4663\\ 0.5337 \end{array}\right),\left(\begin{array}{c} 0\\ 0\\ 0.1923\\ 0.8077 \end{array}\right).$$

*The resulting $F^{\prime }$ is considerably larger than F: one way to see this is that, for any of these vertices P, one has $D_{2}(\hat{P}\left||P\right)=\infty$. This example shows the importance of working with the set F, rather than with just its projections $F_{U|s}$.*

## 5. Polyhedral Approximation: PolyOpt

In this section, we introduce *PolyOpt*, a family of mechanisms Q with good utility obtained by enclosing F by a polyhedron, and then using robust optimization for polyhedra [16] to describe the space of possible Q as a polyhedron; we then maximize the mutual information over this polyhedron. This approach is related to the polyhedral approach of [3], which finds the optimum for this problem in a non-robust setting.

For a mechanism Q and y∈Y, we define $Q_{y}={\left(Q_{y|x}\right)}_{x\in X}\in R^{X}$ to be the *y*-th row of the stochastic matrix *Q* corresponding to Q, but transposed (i.e., viewed as a column vector). Likewise, we define the column vector $Q_{y|s}={\left(Q_{y|s,u}\right)}_{u\in U}\in R^{U}$. In this notation, the condition for (ε,F)-RLDP can be formulated as

(39) $$\forall y\in Y\;\forall s_{1},s_{2}\in S:\max_{P\in F}\left[P_{U|s_{1}}^{T}Q_{y|s_{1}}-e^{\varepsilon }P_{U|s_{2}}^{T}Q_{y|s_{2}}\right]\leq 0.$$

Equation (39) boils down to a set of linear constraints in $Q_{y}$. What makes these difficult to satisfy is that every value P∈F provides a linear constraint, and each $Q_{y}$ has to satisfy all infinitely many of these. In this section, we address this difficulty by making the set F slightly larger, so that robust optimization [16] becomes a convenient tool for optimizing over the allowed Q. More precisely, for every s∈S, let $D_{s}\subset P_{U}$ be such that $F_{U|s}\subset D_{s}$. Then, certainly

(40) $$\max_{P\in F}\left[P_{U|s_{1}}^{T}Q_{y|s_{1}}-e^{\varepsilon }P_{U|s_{2}}^{T}Q_{y|s_{2}}\right]\leq \max_{\begin{array}{c} R_{1}\in D_{s_{1}},\\ R_{2}\in D_{s_{2}} \end{array}}\left[R_{1}^{T}Q_{y|s_{1}}-e^{\varepsilon }R_{2}^{T}Q_{y|s_{2}}\right].$$

Thus, we can conclude that Q is (ε,F)-RLDP whenever

(41) $$\forall y\in Y\;\forall s_{1},s_{2}\in S:\max_{\begin{array}{c} R_{1}\in D_{s_{1}},\\ R_{2}\in D_{s_{2}} \end{array}}\left[R_{1}^{T}Q_{y|s_{1}}-e^{\varepsilon }R_{2}^{T}Q_{y|s_{2}}\right]\leq 0.$$

The trick is now to choose the $D_{s}$ in such a way that the set of Q satisfying (41) has a closed-form description. To this end, we let each $D_{s}$ be a polyhedron; that way, we can use robust optimization for polyhedra [16] to give such a description.

There are multiple ways to create a polyhedron $D_{s}$ that envelops $F_{U|s}$. Writing $L_{u|s}=L_{u|s}\left(F\right)$ for convenience, we take

(42) $$D_{s}=\left\{R\in P_{U}:\forall u\;R_{u}\geq L_{u|s}\right\}.$$

Since $D_{s}$ is described by linear equations, it is a polyhedron, and certainly $F_{U|s}\subset D_{U|s}$ for all *s*. Robust optimization for polytopes [16] then allows us to describe the set of mechanisms satisfying (41). To formulate this, we first need the following definition:

**Definition 3.**  *Let ε>0. Then, define $\Gamma_{\varepsilon }$ to be the convex cone consisting of all $v\in R_{\geq 0}^{X}$ that satisfy, for all $s_{1},s_{2}\in S$ and all $u_{1},u_{2}\in U$:*

(43) $$v_{s_{1},u_{1}}-e^{\varepsilon }v_{s_{2},u_{2}}+\sum_{u}L_{u|s_{1}}\left(v_{s_{1},u}-v_{s_{1},u_{1}}\right)-e^{\varepsilon }\sum_{u}L_{u|s_{2}}\left(v_{s_{2},u}-v_{s_{2},u_{2}}\right)\leq 0.$$

Note that, for every choice of $s_{1},s_{2},u_{1},u_{2}$, (3) is a linear inequality in *T* and thus defines a half-space in $R^{X}$. The intersection of these half-spaces, intersected with $R_{\geq 0}^{X}$, defines the convex cone $\Gamma_{\varepsilon }$. This definition allows us to formulate the following result:

**Theorem 2.**  *Let Q be a privacy mechanism, and for y∈Y, let $Q_{y}$ be the y-th row of the associated matrix $Q={\left(Q_{y|x}\right)}_{y\in Y,x\in X}$. Suppose that for all y we have $Q_{y}\in \Gamma_{L}$. Then, Q satisfies (ε,F)-RLDP.*

The upshot of this theorem is that we have translated the infinitely many constraints of (39) and (41) into the finitely many linear constraints of (3). This makes optimizing utility considerably easier. We perform this optimization by translating it into a linear programming problem. The key inspiration for this optimization is Theorem 4 of [5], where optimal LDP mechanisms are found by translating the problem of optimizing mutual information into linear programming; we use an analogous approach adapted to RLDP. This approach can be sketched as follows: Let $\hat{\Gamma }=\left\{v\in \Gamma_{\varepsilon }:\sum_{x}v_{x}=1\right\}$, i.e., the intersection of $\Gamma_{\varepsilon }$ with the hyperplane corresponding to $\sum_{x}v_{x}=1$. This is a polyhedron, and every Q satisfying the conditions of Theorem 2 has $Q_{y}=\theta_{y}v_{y}$, for some $\theta_{y}\in R_{\geq 0}$ and $v_{y}\in \hat{\Gamma }$. The authors of [5] made a number of key observations that also apply to our situation. The first is that, in this case, we can write

(44) $$I_{\hat{P}}\left(X;Y\right)=\sum_{y}\theta_{y}\mu \left(v_{y}\right),$$

where

(45) $$\mu \left(v\right)=\sum_{x\in X}v_{x}{\hat{P}}_{x}\log\frac{v_{x}}{\sum_{x^{\prime }}v_{x^{\prime }}{\hat{P}}_{x^{\prime }}}.$$

The second observation is that, in order to maximize (44), one can prove from the convexity of μ that it is optimal to have each $v_{y}$ be a vertex of $\hat{\Gamma }$. Thus, once we know the set of vertices V of $\hat{\Gamma }$, we find the optimal Q by assigning a weight $\theta_{v}$ to each v∈V, in such a way that the resulting $Q_{y}$ form a probabilistic matrix and such that (44) is maximized. Since (44) is linear in θ, this is a linear programming problem. This discussion is summarized in the following theorem:

**Theorem 3.**  *Let $\hat{\Gamma }$ be a polyhedron given by $\left\{v\in \Gamma_{L,\varepsilon }:\sum_{x}v_{x}=1\right\}$. Let V be the set of vertices of $\hat{\Gamma }$. Define μ as in (45). Let $1_{X}\in R^{X}$ be the constant vector of ones. Let $\hat{\theta }\in R_{\geq 0}^{V}$ be the solution to the optimization problem*

(46) $$\begin{array}{cc} {\mathrm{maximise}}_{\theta } & \;\sum_{v\in V}\theta_{v}\mu \left(v\right)\\ \mathrm{satisfying} & \;\theta \in R_{\geq 0}^{V},\\ \; & \;\sum_{v\in V}\theta_{v}v=1_{X}. \end{array}$$

*Let the privacy mechanism Q be given by $Y=\{v\in V:{\hat{\theta }}_{v}>0\}$ and $Q_{v|x}={\hat{\theta }}_{v}v_{x}$. Then, the mechanism Q maximizes $I_{\hat{P}}\left(X;Y\right)$ among all mechanisms satisfying the condition of Theorem 2. One has $\left|Y\right|\leq a$.*

Together, Theorems 2 and 3 show that if we can solve a vertex enumeration problem, we can find a mechanism Q that maximizes $I_{\hat{P}}\left(X;Y\right)$ among a subset of all (ε,F)-RLDP mechanisms; furthermore, we ensure that the output space Y is, at most, the size of the input space X. The proof of Theorem 3 is analogous to the proof of Theorem 4 of [5] and is given in Appendix A.5. Note that the results of [5] do not run into the vertex enumeration problem, because the relevant polyhedron there is ${\left[1,e^{\varepsilon }\right]}^{X}$, for which the vertices are known.

We remark that a simplex is not the only possible choice for $D_{s}$. In general, we can make $D_{s}$ closer to $F_{U|s}$ by adding more defining hyperplanes. Doing this allows more Q to satisfy Theorem 2 and in turn increases the utility of the Q we find via Theorem 3. However, since Γ is related to the $D_{s}$ via duality, adding extra constraints to the $D_{s}$ will increase the dimension of Γ through the addition of auxiliary variables. This makes the vertex enumeration problem of Theorem 3 more computationally involved. Thus, we have a trade-off between utility and computational complexity. Even with the given, ‘simple’ choice of $D_{s}$, the computational complexity is quite high: recall that we defined a=|X|. The polytope $\hat{\Gamma }$ is (a−1)-dimensional and is defined by $a^{2}+a$ inequalities, thus it has $O\left({\left(a^{2}+a\right)}^{\frac{a-1}{2}}\right)=O\left(a^{a}\right)$ vertices [58]. Since this is the dimension of the linear programming problem, we find that the total complexity of finding Q is $O(a^{\omega a}\log\left(a^{a+1}/\delta \right))$, where ω≈2.38 is the exponent of matrix multiplication and δ the relative accuracy [58]. Clearly, this becomes infeasible rather quickly for large *a*.

It should be noted that, in general, the increasing utility obtained by decreasing $D_{s}$ in size does not approach the optimal utility over all (ε,F)-RLDP mechanisms. This is because, as we take increasingly finer $D_{U|s}$, we approach the set of Q that satisfy (4) for all *P* in $F^{\prime }:=\left\{P:\forall s\;P_{U|s}\in F_{U|s}\right\}$. As discussed in Section 4.1, one has $F⊊F^{\prime }$. As a result, the set of $\left(\varepsilon ,F^{\prime }\right)$-RLDP mechanisms is strictly smaller than the set of (ε,F)-RLDP mechanisms.

**Example 3.**  *We continue Example 2 by taking ε=log2. To obtain $\hat{\Gamma }$ in Theorem 3, we need to combine the defining inequalities of $\Gamma_{\varepsilon }$ in Definition 3, along with the defining equality $\sum_{x}P_{x}=1$. Regarding the inequalities, we have $2^{4}=16$ inequalities of the form (3), as well as 4 inequalities of the form $v_{x}\geq 0$. Together with the equality constraint, we obtain a 3-dimensional polytope in $R^{X}=R^{4}$. Using a vertex enumeration algorithm, one finds that V consists of the rows of the matrix V below, where the order of the columns is the order of the rows of Example 1. For each row v, we can calculate μ(v), resulting in the vector μ below. Solving (46), we obtain the vector $\hat{\theta }$ below:*

(47) $$V=\left(\begin{array}{cccc} 0.0744 & 0.3227 & 0.5603 & 0.0426\\ 0.2426 & 0.2426 & 0.4783 & 0.0364\\ 0.3333 & 0.3333 & 0.1667 & 0.1667\\ 0.1091 & 0.4737 & 0.2086 & 0.2086\\ 0.0993 & 0.4310 & 0 & 0.4697\\ 0.1121 & 0.4864 & 0 & 0.4015\\ 0.3404 & 0.3404 & 0 & 0.3191\\ 0.0770 & 0.3343 & 0.2944 & 0.2944\\ 0.2234 & 0.2234 & 0 & 0.5531\\ 0.4875 & 0.1434 & 0 & 0.3690\\ 0.4360 & 0.1283 & 0 & 0.4358\\ 0.4758 & 0.1400 & 0.1921 & 0.1921\\ 0.3437 & 0.1011 & 0.2776 & 0.2776\\ 0.1602 & 0.1602 & 0.6316 & 0.0481\\ 0.1667 & 0.1667 & 0.3333 & 0.3333\\ 0.3325 & 0.0978 & 0.5294 & 0.0403 \end{array}\right),\mu =\left(\begin{array}{c} 0.1152\\ 0.0942\\ 0.0087\\ 0.0135\\ 0.1097\\ 0.0968\\ 0.0723\\ 0.0080\\ 0.1240\\ 0.0878\\ 0.1014\\ 0.0106\\ 0.0076\\ 0.1240\\ 0.0075\\ 0.1083 \end{array}\right),\hat{\theta }=\left(\begin{array}{c} 1.1899\\ 0\\ 0\\ 0\\ 0\\ 0.7670\\ 0\\ 0\\ 0\\ 0\\ 1.4134\\ 0\\ 0\\ 0\\ 0\\ 0.6297 \end{array}\right).$$

*We now obtain the privacy mechanism $Q_{\mathrm{PolyOpt}}$ as follows: each row of $Q_{\mathrm{PolyOpt}}$ corresponds to a non-zero coefficient of $\hat{\theta }$, multiplied by its corresponding row of V. Thus, we obtain*

(48) $$\begin{array}{cc} Q_{\mathrm{PolyOpt}} & =\left(\begin{array}{cccc} Q_{y_{1}|s_{1},u_{1}} & Q_{y_{1}|s_{1},u_{2}} & Q_{y_{1}|s_{2},u_{1}} & Q_{y_{1}|s_{2},u_{2}}\\ Q_{y_{2}|s_{1},u_{1}} & Q_{y_{2}|s_{1},u_{2}} & Q_{y_{2}|s_{2},u_{1}} & Q_{y_{2}|s_{2},u_{2}}\\ Q_{y_{3}|s_{1},u_{1}} & Q_{y_{3}|s_{1},u_{2}} & Q_{y_{3}|s_{2},u_{1}} & Q_{y_{3}|s_{2},u_{2}}\\ Q_{y_{4}|s_{1},u_{1}} & Q_{y_{4}|s_{1},u_{2}} & Q_{y_{4}|s_{2},u_{1}} & Q_{y_{4}|s_{2},u_{2}} \end{array}\right) \end{array}$$

(49) $$\begin{array}{cc} \; & =\left(\begin{array}{cccc} 0.0885 & 0.3840 & 0.6667 & 0.0507\\ 0.0860 & 0.3731 & 0 & 0.3080\\ 0.6162 & 0.1813 & 0 & 0.6159\\ 0.2094 & 0.0616 & 0.3333 & 0.0254 \end{array}\right). \end{array}$$

Note that indeed we have 4=b≤a=4. As for the utility, we have $I_{\hat{P}}\left(X;Y\right)=\mu \cdot \hat{\theta }=0.4228$. However, the true utility is significantly lower, namely $I_{P^{*}}\left(X;Y\right)=0.2804$.

## 6. An Optimal Policy for $F=P_{X}$

As PolyOpt mechanisms are obtained via vertex enumeration in *a*-dimensional space, this can be computationally infeasible for larger *a*. Thus, there is a need for methods that, given $\hat{P}$ and F, can find (ε,F)-RLDP mechanisms with reasonable computational complexity.

In this section, we consider the case where F is maximal, i.e., $F=P_{X}$. By itself, this represents a situation where we want privacy for every possible probability distribution on X. This scenario may not be very relevant in practice, but any protocol that we in find this way is also (ε,F)-RLDP for *any* F. As we will see below, this allows us to find (ε,F)-RLDP protocols in a computationally efficient manner.

We show that $\left(\varepsilon ,P_{X}\right)$-RLDP is almost equivalent to LDP. We exploit this to create SRR, the RLDP analogue to GRR [5], the LDP mechanism that is optimal for ε≫0. SRR only depends on ε and X and not on $\hat{P}$, and as such does not require an optimization procedure to be found; this makes it a good choice when vertex enumeration is computationally infeasible. The downside is that SRR has a stricter privacy requirement than PolyOpt, as it takes F to be maximal; in Section 8, we investigate numerically to what extent this results in a lower utility.

We start by giving a characterization of $\left(\varepsilon ,P_{X}\right)$-RLDP. Like LDP, this can be defined by an inequality constraint on the matrix *Q*.

**Proposition 3.**  *Q satisfies $\left(\varepsilon ,P_{X}\right)$-RLDP if and only if for all y∈Y and $\left(s,u\right),\left(s^{\prime },u^{\prime }\right)\in X$ with $s\neq s^{\prime }$ one has*

(50) $$\frac{Q_{y|s,u}}{Q_{y|s^{\prime },u^{\prime }}}\leq e^{\varepsilon }.$$

**Proof.**  Suppose that Q satisfies (ε,F)-RLDP with respect to $P_{X}$. Let $\left(s,u\right),\left(s^{\prime },u^{\prime }\right)\in X$ with $s\neq s^{\prime }$. Let *P* be given by

(51) $$P_{x}=\left\{\begin{array}{cc} \frac{1}{2},\; & \mathrm{if}\;x\in \{\left(s,u\right),\left(s^{\prime },u^{\prime }\right)\},\\ 0,\; & \mathrm{otherwise}. \end{array}\right.$$

Then, $P_{u|s}=1$ and $P_{u^{\prime \prime }|s}=0$ for all $u^{\prime \prime }\neq u$; an analogous statements holds for $P_{u^{\prime }|s^{\prime }}$. It follows that

(52)Qy|s,uQy|s′,u′=Qy|s,uPu|sQy|s′,u′Pu′|s′(53)=∑u″Qy|s,u″Pu″|s∑u″Qy|s′,u″Pu″|s′(54)=PX∼P(Q(X)=y|S=s)PX∼P(Q(X)=y|S=s′)≤eε.

This proves “⇒”. On the other hand, suppose that $\frac{Q_{y|s,u}}{Q_{y|s^{\prime },u^{\prime }}}\leq e^{\varepsilon }$ for all $s\neq s^{\prime }$ and $u,u^{\prime }$. Then, for all $s\neq s^{\prime }$ and *P*, we have

(55) $$\frac{P_{X∼P}\left(Q\left(X\right)=y|S=s\right)}{P_{X∼P}\left(Q\left(X\right)=y|S=s^{\prime }\right)}=\frac{\sum_{u}Q_{y|s,u}P_{u|s}}{\sum_{u^{\prime }}Q_{y|s^{\prime },u^{\prime }}P_{u^{\prime }|s^{\prime }}}\leq e^{\varepsilon }.$$

Hence, Q satisfies $\left(\varepsilon ,P_{X}\right)$-RLDP with respect to. F. □

The proposition demonstrates that RLDP is very similar to LDP. The difference is that the condition “for all $x,x^{\prime }\in X$” from Definition 2 is relaxed to only those *x* and $x^{\prime }$ for which $s\neq s^{\prime }$.

Before moving on and introducing a new mechanism, note that Proposition 3 clearly illustrates the reason that the setting in this paper cannot be modeled using the block-structured approach from [12]. We see that if $u\neq u^{\prime }$, we still have a privacy constraint, whereas in [12] this is not the case.

Next, we will introduce a mechanism that exploits the difference between LDP and RLDP. Recall that a=|X|; then generalized randomized response [19] is the privacy mechanism ${\mathrm{GRR}}^{\varepsilon }:X\to X$ given by

(56) $${\mathrm{GRR}}_{y|x}^{\varepsilon }=\left\{\begin{array}{cc} \frac{e^{\varepsilon }}{e^{\varepsilon }+a-1} & \mathrm{if}\;x=y,\\ \frac{1}{e^{\varepsilon }+a-1} & \mathrm{otherwise}. \end{array}\right.$$

This mechanism has been designed such that $\frac{{\mathrm{GRR}}_{y|x}^{\varepsilon }}{{\mathrm{GRR}}_{y|x^{\prime }}^{\varepsilon }}=e^{\pm \varepsilon }$ for $x\neq x^{\prime }$, the maximal fractional difference that ε-LDP allows. We will see that for RLDP we can go up to a difference of $e^{\pm 2\varepsilon }$ if x=(s,u) and $x^{\prime }=\left(s,u^{\prime }\right)$, as we typically only need to satisfy

(57) $$Q_{y|s,u}\leq e^{\varepsilon }Q_{y|s^{\prime },u^{\prime }}\leq e^{2\varepsilon }Q_{y|s,u^{\prime }}.$$

We capture the intuition from the necessary condition (57) in a new mechanism called *secret randomized response (SRR)*. Recall that $a_{1}=\left|S\right|$, $a_{2}=\left|U\right|$.

**Definition 4.**  (Secret randomized response (SRR))**.** *Let ε>0. Then, the privacy mechanism ${\mathrm{SRR}}^{\varepsilon }:X\to X$ is given by*

(58) $${\mathrm{SRR}}_{s^{\prime },u^{\prime }|s,u}^{\varepsilon }=\left\{\begin{array}{cc} \frac{e^{\varepsilon }}{e^{\varepsilon }+e^{-\varepsilon }\left(a_{2}-1\right)+a-a_{2}}, & if\;\left(s^{\prime },u^{\prime }\right)=\left(s,u\right),\\ \frac{e^{-\varepsilon }}{e^{\varepsilon }+e^{-\varepsilon }\left(a_{2}-1\right)+a-a_{2}}, & if\;s^{\prime }=s\;and\;u^{\prime }\neq u,\\ \frac{1}{e^{\varepsilon }+e^{-\varepsilon }\left(a_{2}-1\right)+a-a_{2}}, & if\;s^{\prime }\neq s, \end{array}\right.$$

It is clear that $\frac{{\mathrm{SRR}}_{y|s,u}^{\varepsilon }}{{\mathrm{SRR}}_{y|s^{\prime },u^{\prime }}^{\varepsilon }}\in \left\{e^{-2\varepsilon },e^{\varepsilon },1,e^{\varepsilon },e^{2\varepsilon }\right\}$, and the two extreme cases are only possible when $s=s^{\prime }$. Thus, we can conclude

**Lemma 2.**  *SRR satisfies $\left(\varepsilon ,P_{X}\right)$-RLDP.*

**Example 4.**  *We continue Example 3. Although SRR is closely related to GRR, adopting it can still have a significant impact on utility. For instance, in the setting of Example 3, we obtain*

(59) $${\mathrm{GRR}}^{\varepsilon }=\left(\begin{array}{cccc} 0.4 & 0.2 & 0.2 & 0.2\\ 0.2 & 0.4 & 0.2 & 0.2\\ 0.2 & 0.2 & 0.4 & 0.2\\ 0.2 & 0.2 & 0.2 & 0.4 \end{array}\right),\;\;{\mathrm{SRR}}^{\varepsilon }=\left(\begin{array}{cccc} 0.444 & 0.111 & 0.222 & 0.222\\ 0.111 & 0.444 & 0.222 & 0.222\\ 0.222 & 0.222 & 0.444 & 0.111\\ 0.222 & 0.222 & 0.111 & 0.444 \end{array}\right).$$

*Then,*

(60) $$\begin{array}{cccc} I_{\hat{P}}\left(X;{\mathrm{GRR}}^{\varepsilon }\left(X\right)\right) & =0.0419, & \;\;\;\;\;\;\;\;I_{\hat{P}}\left(X;{\mathrm{SRR}}^{\varepsilon }\left(X\right)\right) & =0.1005, \end{array}$$

(61) $$\begin{array}{cccc} I_{P^{*}}\left(X;{\mathrm{GRR}}^{\varepsilon }\left(X\right)\right) & =0.0412, & \;\;\;\;\;\;\;\;I_{P^{*}}\left(X;{\mathrm{SRR}}^{\varepsilon }\left(X\right)\right) & =0.0942. \end{array}$$

*We see that adopting SRR more than doubles the utility. Compared to Example 3, we see that the utility is still significantly lower than that of PolyOpt, but the advantage is that we obtain SRR directly from ε, without having to take $\hat{P}$ or F into account; this ensures a significantly faster computation.*

The power of SRR, beyond slightly improving on GRR, is that we can prove it maximizes $I_{P}\left(X;Y\right)$ for sufficiently large ε; the cutoff point depends on *P*. This is proven analogously to the result of [5], where GRR is the optimal LDP mechanism for sufficiently large ε.

**Theorem 4.**  *For every P, there is an $\varepsilon_{0}\geq 0$ such that for all $\varepsilon \geq \varepsilon_{0}$, SRR is the $\left(\varepsilon ,P_{X}\right)$-RLDP mechanism maximizing $I_{P}\left(X;Y\right)$.*

The proof of this theorem follows the same lines as the proof of Theorem 14 of [5], in which it is proven that GRR is the optimal LDP mechanism for sufficiently large ε. The proof is presented in Appendix A.6. This solves the problem of finding the optimal $\left(\varepsilon ,P_{X}\right)$-mechanism, for sufficiently large ε. This strategy is similar to the proof of Theorem 3: one can show that the rows $Q_{y}$ of the optimal (ε,F)-RLDP mechanism Q correspond to vertices of a polyhedron, and the optimal weights assigned to these vertices are found using a linear programming problem. Unlike in the case of Theorem 3, however, we can give an explicit description of the set of vertices, and we can solve the linear programming problem analytically.

Our result shows that if one wishes to satisfy $\left(\varepsilon ,P_{X}\right)$-RLDP, then SRR is a solid choice, especially for larger ε, since it maximizes $I_{P^{*}}\left(X;Y\right)$ for sufficiently ε. Thus, we can optimize $I_{P^{*}}\left(X;Y\right)$ without having to know $P^{*}$, with the caveat that the cutoff point for ‘large enough’ depends on $P^{*}$.

In [5], the optimal LDP mechanism in the high-privacy regime (i.e., ε≪1) was also found. In principle, we could also do this for $\left(\varepsilon ,P_{X}\right)$-RLDP, but this would not be of much use, as the optimal mechanism would depend on $P^{*}$, which we assume to be unknown.

## 7. Independent Reporting

Section 5 demonstrated the need to find efficiently computable (ε,F)-RLDP mechanisms with decent utility. In Section 6, we approach this problem by considering $\left(\varepsilon ,P_{X}\right)$-RLDP instead, allowing us to analytically obtain the optimal mechanism. However, when F is small, this overapproximation might result in a large loss of utility. In this section, we describe *independent reporting* (IR), a different heuristic that takes the size of F into account, while still being significantly less computationally complex than PolyOpt.

The basis of IR is to apply two separate LDP mechanisms $R^{1}$ and $R^{2}$ to *S* and *U*, respectively, reporting both outputs.

**Definition 5.**  *Let $Y^{1},Y^{2}$ be sets, and let $Y=Y^{1}\times Y^{2}$. Let $R^{1}:S\to Y^{1}$ and $R^{2}:U\to Y^{2}$ be probabilistic maps. Then, theindependent reporting of $R^{1}$ and $R^{2}$ is the probabilistic map ${IR}_{R^{1},R^{2}}:X\to Y$ given by ${IR}_{R^{1},R^{2}}\left(s,u\right)=\left(R^{1}\left(s\right),R^{2}\left(u\right)\right)$.*

Suppose that $R^{i}$ satisfies $\varepsilon_{i}$-LDP. The composition theorem for differential privacy [59] tells us that ${IR}_{R^{1},R^{2}}$ satisfies $\left(\varepsilon_{1}+\varepsilon_{2}\right)$-LDP. However, in the RLDP setting, *U* only indirectly leaks information about *S*; therefore, we can get away with a higher $\varepsilon_{2}$ compared to the LDP setting. How much higher depends on the degree of relatedness of *S* and *U*, which is captured by the possible values of *P* in F. The precise statement is given in the following result:

**Theorem 5.**  *Let $\varepsilon_{1},\varepsilon_{2}\in R_{\geq 0}$. For each s, let $d_{s}\in \left[0,\infty \right)$ be such that $d_{s}\geq {\mathrm{rad}}_{s}\left(F\right)$. Furthermore, define*

(62) $$d=\min\left\{2,\max_{s}\left(2d_{s}\right)+\max_{s,s^{\prime }}\left|\right|{\hat{P}}_{U|s}-{\hat{P}}_{U|s^{\prime }}{\left|\right|}_{1}\right\}.$$

*Let $\delta_{2}=\log\left(1+\frac{2(e^{\varepsilon_{2}}-1)}{d}\right)$. Suppose that $R^{1}$ is $\varepsilon_{1}$-LDP and that $R^{2}$ is $\delta_{2}$-LDP. Then, IR is $\left(\varepsilon_{1}+\varepsilon_{2},F\right)$-RLDP.*

If S=U, then $\left|\right|{\hat{P}}_{U|s}-{\hat{P}}_{U|s^{\prime }}{\left|\right|}_{1}=2$ for $s\neq s^{\prime }$, so d=2 and $\delta_{2}=\varepsilon_{2}$. In this case, Theorem 5 is the RLDP analogue to the well-known composition theorem for local differential privacy [59]. In general, $\delta_{2}\geq \varepsilon_{2}$; this represents the fact the privacy requirement on $R^{2}$ is less strict when *S* and *U* are only partially related. At the other extreme, if *S* and *U* are independent in our observation, we have $\left|\right|{\hat{P}}_{U|s}-{\hat{P}}_{U|s^{\prime }}{\left|\right|}_{1}=0$ for all $s,s^{\prime }$. Still, we cannot fully disclose *U*, since *S* and *U* might be non-independent under $P^{*}$. The term $d_{s}$ is present in the definition of *d* to account for this possibility.

In order to prove Theorem 5, we need the following lemma:

**Lemma 3.**  *Let Q:X→Y be an ε-LDP mechanism. Then, for all y∈Y and all $P,P^{\prime }\in P_{X}$ we have*

(63) $$\frac{P_{X∼P}\left(Q\left(X\right)=y\right)}{P_{X∼P^{\prime }}\left(Q\left(X\right)=y\right)}\leq 1+\frac{e^{\varepsilon }-1}{2}||P-P^{\prime }{\left|\right|}_{1}.$$

**Proof.**  Fix *y*, and let $Q_{y}^{\max}=\max_{x}Q_{y|x}$ and $Q_{y}^{\min}=\min_{x}Q_{y|x}$. By the ε-LDP property, it holds that $Q_{y}^{\max}\leq e^{\varepsilon }Q_{y}^{\min}$. We hence find

(64)PX∼P(Q(X)=y)−PX∼P′(Q(X)=y)=∑x∈XQy|x(Px−Px′)(65)=∑x:Px≥Px′Qy|x(Px−Px′)−∑x:Px′>PxQy|x(Px′−Px)(66)≤Qymax2||P−P′||1−Qymin2||P−P′||1(67)≤(eε−1)Qymin2||P−P′||1(68)≤(eε−1)PX∼P′(Q(X)=y)2||P−P′||1,

from which the lemma directly follows. □

**Proof**  (Proof of Theorem 5)**.** We start by showing that *d* is an upper bound for $\left|\right|P_{U|s}-P_{U|s^{\prime }}{\left|\right|}_{1}$. If d=2, this is certainly the case. Suppose $d=\max_{s}\left(2d_{s}\right)+\max_{s,s^{\prime }}\left|\right|{\hat{P}}_{U|s}-{\hat{P}}_{U|s^{\prime }}{\left|\right|}_{1}$. Then, for all $s,s^{\prime }\in S$ and P∈F we have

(69)||PU|s−PU|s′||1≤||PU|s−P^U|s||1+||P^U|s−P^U|s′||1+||P^U|s′−PU|s′||1(70)≤ds+ds′+||P^U|s−P^U|s′||1(71)≤d.

Combining Lemma 3 with the fact that $\varepsilon_{2}=\log\left(1+\frac{d(e^{\delta_{2}}-1)}{2}\right)$, it follows that for every $y_{2}\in Y_{2}$, we have

(72)PX∼P(R2(U)=y2|S=s)PX∼P(R2(U)=y2|S=s′)≤1+eδ2−12||PU|s−PU|s′||1(73)≤1+d(eδ2−1)2(74)=eε2.

Given *S*, the random variables $R^{1}\left(S\right)$ and $R^{2}\left(U\right)$ are independent. It follows that for every $y_{1}\in Y_{1}$ and every $y_{2}\in Y_{2}$, we have

(75)P(R1(S)=y1,R2(U)=y2|S=s)P(R1(S)=y1,R2(U)=y2|S=s′)=P(R1(S)=y1,|S=s)P(R1(S)=y1|S=s′)·P(R2(U)=y2|S=s)P(R2(U)=y2|S=s′)(76)≤eε1+ε2,

where the last equality holds because of (74) and because $R^{1}$ is $\varepsilon_{1}$-LDP. This shows that ${IR}_{R^{1},R^{2}}$ is $\left(\varepsilon_{1}+\varepsilon_{2},F\right)$-RLDP. □

Theorem 5 establishes the privacy of independent reporting. To maximize the utility, we need to determine how to divide the privacy budget ε between $\varepsilon_{1}$ and $\varepsilon_{2}$, and which LDP mechanisms to use for $R^{1}$ and $R^{2}$. To answer both these questions, we first need an expression for the utility of IR, which is given by the following theorem:

**Theorem 6.**  *For any $P\in P_{X}$, one has*

(77) $$I_{P}\left({IR}_{R^{1},R^{2}}\left(X\right);X\right)=I_{P}\left(R^{1}\left(S\right);S\right)+I_{P}\left(R^{2}\left(U\right);U|R^{1}\left(S\right)\right).$$

**Proof.**  Since $R^{1}\left(S\right)$ and *U* are independent given *S*, and $R^{2}\left(U\right)$ and *S* are independent given *U* and $R^{1}\left(S\right)$, we have

(78)IP(IRR1,R2(X);X)=IP(R1(S),R2(U);U,S)(79)=IP(R1(S);U,S)+IP(R2(U);U,S|R1(S))(80)=IP(R1(S);S)+IP(R2(U);U|R1(S)).

□

We use Theorems 5 and 6 to find high-utility IR protocols that satisfy (ε,F)-RLDP, given ε and F. To do so, we need to choose $R^{1}$ and $R^{2}$, and split the privacy budget between them. Since the expression for the utility of IR in Theorem 6 contains a term $I_{P}\left(R^{1}\left(S\right);S\right)$, the $R^{1}$ that maximizes this is GRR when ε is large enough; thus, we choose $R^{1}=\mathrm{GRR}$. The second term in the utility expression is

(81) $$I_{P}\left(R^{2}\left(U\right);U|R^{1}\left(S\right)\right)=E_{r}\left[I_{U∼P_{U}|R^{1}\left(S\right)=r}\left(R^{2}\left(U\right);U\right)\right].$$

This is the expected value of an expression that is maximized for $R^{2}=\mathrm{GRR}$, with the caveat that the maximization only holds when ε is large enough, and what ‘large enough’ is depends on the distribution of *U*. Since this gives us a choice of $R^{2}$ independent of the distribution, we ignore this caveat and take $R^{2}=\mathrm{GRR}$ as well.

Having chosen $R^{1}$ and $R^{2}$, we are only left with the division of the privacy budget. If we choose $\varepsilon_{2}$, then by Theorem 5 the privacy parameters of $R^{1}$ and $R^{2}$ are $\varepsilon_{1}=\varepsilon -\varepsilon_{2}$ and $\delta_{2}=\log\left(1+\frac{2(e^{\varepsilon_{2}}-1)}{d}\right)$, respectively. It follows that to find a high-utility IR protocol, we have to solve the following optimization problem:

(82) $$\begin{array}{cc} {\mathrm{maximize}}_{\varepsilon_{2}}\; & I_{P}\left({\mathrm{GRR}}_{\varepsilon -\varepsilon_{2}}\left(S\right),{\mathrm{GRR}}_{\log\left(1+\frac{2(e^{\varepsilon_{2}}-1)}{d}\right)}\left(U\right);S,U\right)\\ \mathrm{subject}\;\mathrm{to}\; & \varepsilon_{2}\in \left[0,\varepsilon \right]. \end{array}$$

This optimization problem is only 1-dimensional. While it is not straightforward to express the complexity of solving this in O-notation, our experiments in Section 8 show this can be quickly performed numerically, and significantly faster than PolyOpt.

**Example 5.**  *We continue Example 4. Having found ${\mathrm{rad}}_{s}\left(F\right)$ and ${\hat{P}}_{U|s_{1}},{\hat{P}}_{U|s_{2}}$ in Example 2, we conclude that, in Theorem 5, we have*

(83) d=min2,2·max{0.6107,0.3061}+0.41180.5882−0.31330.68671=1.4591.

*It follows that $\delta_{2}=\log\left(1+\frac{2}{1.4591}\left(e^{\varepsilon_{2}}-1\right)\right)=\log\left(1.3707e^{\varepsilon_{2}}-0.3707\right)$. For a given value of $\varepsilon_{2}$, the matrix corresponding to $IR({\mathrm{GRR}}^{\log\left(2\right)-\varepsilon_{2}},{\mathrm{GRR}}^{\delta_{2}})$ is the Kronecker product*

(84) $$\begin{array}{c} \left(\begin{array}{c} \frac{2e^{-\varepsilon_{2}}}{2e^{-\varepsilon_{2}}+1}\frac{1}{2e^{-\varepsilon_{2}}+1}\\ \frac{1}{2e^{-\varepsilon_{2}}+1}\frac{2e^{-\varepsilon_{2}}}{2e^{-\varepsilon_{2}}+1} \end{array}\right)⊗\left(\begin{array}{c} \frac{1.3707e^{\varepsilon_{2}}-0.3707}{1.3707e^{\varepsilon_{2}}+0.6293}\frac{1}{1.3707e^{\varepsilon_{2}}+0.6293}\\ \frac{1}{1.3707e^{\varepsilon_{2}}+0.6293}\frac{1.3707e^{\varepsilon_{2}}-0.3707}{1.3707e^{\varepsilon_{2}}+0.6293} \end{array}\right)\\ =\frac{1}{C}\left(\begin{array}{cccc} 2.7414-0.7414e^{-\varepsilon_{2}} & 2e^{-\varepsilon_{2}} & 1.3707e^{\varepsilon_{2}}-0.3707 & 1\\ 2e^{-\varepsilon_{2}} & 2.7414-0.7414e^{-\varepsilon_{2}} & 1 & 1.3707e^{\varepsilon_{2}}-0.3707\\ 1.3707e^{\varepsilon_{2}}-0.3707 & 1 & 2.7414-0.7414e^{-\varepsilon_{2}} & 2e^{-\varepsilon_{2}}\\ 1 & 1.3707e^{\varepsilon_{2}}-0.3707 & 2e^{-\varepsilon_{2}} & 2.7414-0.7414e^{-\varepsilon_{2}} \end{array}\right), \end{array}$$

*where $C=\left(2e^{-\varepsilon_{2}}+1\right)\left(1.3707e^{\varepsilon_{2}}+0.6293\right)$. We now wish to optimize its utility, i.e., find the $\varepsilon_{2}\in \left[0,\log2\right]$ that maximizes $I_{\hat{P}}\left(X;Y\right)$. The optimum occurs at the boundary $\varepsilon_{2}=\log\left(2\right)$, for which $I_{\hat{P}}\left(X;Y\right)=0.0755$. Notice that now $\varepsilon_{1}=0$, so $R^{1}={\mathrm{GRR}}^{0}$ is completely random: its output does not depend on the input. In other words, the optimal IR protocol in this case does not transmit any direct information about S at all, only indirectly through ${\mathrm{GRR}}^{\delta_{2}}\left(U\right)$. In this case, we have*

(85) $$Q_{IR}=\left(\begin{array}{cccc} 0.3517 & 0.1483 & 0.3517 & 0.1483\\ 0.1483 & 0.3517 & 0.1483 & 0.3517\\ 0.3517 & 0.1483 & 0.3517 & 0.1483\\ 0.1483 & 0.3517 & 0.1483 & 0.3517 \end{array}\right).$$

Regarding the ‘true’ utility, we have $I_{P^{*}}\left(X;Y\right)=0.0718$. Interestingly, $Q_{IR}$ yields less utility than SRR. As we will see in Section 8, this is typical for small S and U.

## 8. Experiments

In order to gain insight into the behavior of the different mechanisms, we performed several experiments, both on synthetic and real data. We compared the three mechanisms introduced in this paper (PolyOpt, SRR, and IR). Throughout, we let F be a confidence set for a $\chi^{2}$-test, i.e., for a Rényi divergence with α=2. We used the results of Section 4 to find explicit expressions for $L_{u|s}\left(F\right)$ and (an upper bound for) ${\mathrm{rad}}_{s}\left(F\right)$. Recall from Section 3 that

(86) $$F=\left\{P\in P_{X}:D_{2}(\hat{P}\left||P\right)\leq \log\left(1+\frac{F_{\chi^{2},a-1}^{-1}\left(1-\beta \right)}{n}\right)\right\},$$

where $F_{\chi^{2},a-1}$ is the cumulative density function of the $\chi^{2}$-distribution with a−1 degrees of freedom, and β∈(0,1) is a chosen significance level. Throughout the experiments, we took β=0.05, unless otherwise specified.

We used $I_{\hat{P}}\left(X;Y\right)$ as a utility metric, divided by H(X) to obtain the normalized mutual information (NMI). We used this rather than $I_{P^{*}}\left(X;Y\right)$, as the aggregator only has access to the former. In fact, while $P^{*}$ is known for the synthetic data, this is not the case for real data, so we cannot even use $I_{P^{*}}\left(X;Y\right)$ as a utility metric.

We compared our methods to two existing approaches, each with a slightly different privacy model. First, we compared to an LDP mechanism, to see to what extent the RLDP framework offered a utility improvement over regular LDP. As the LDP mechanism, we chose GRR, because it optimizes $I_{P}\left(X;Y\right)$, our privacy metric, in the low-privacy regime [5]. Second, we compared to the non-robust optimal mechanism of [3]. This mechanism is obtained in a manner similar to PolyOpt, and is the optimal mechanism that satisfies (in our notation) $\left(\varepsilon ,\left\{\hat{P}\right\}\right)$-RLDP. In other words, it is optimal in the scenario where one knows $P^{*}$ precisely. We shall refer to this mechanism as NR (non-robust). Typically, we would expect NR to have a higher utility than our RLDP mechanisms, (because it only needs to satisfy privacy with respect to. one distribution) and GRR to have worse a utility (because LDP is stricter than RLDP).

### 8.1. Adult Data Set

**Figure 2:**
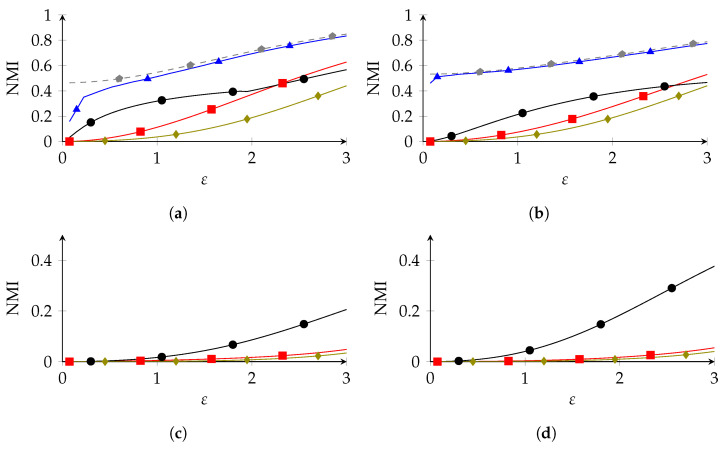
Experiments on the categories *sex*, *race*, *education*, *occupation*, *relationship* and *native-country* of the adult data set. Numbers between brackets indicate $a_{1}$ and $a_{2}$ (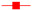 SRR, 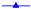 PolyOpt, 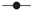 IR, 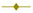 GRR, 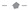 NR). (**a**) S= sex (2), U= race (5), (**b**) S= race (5), U= sex (2), (**c**) S= occ. (15), U= edu. (16), (**d**) S= native country (42), U= relationship (6).

We performed numerical experiments on the adult data set (*n* = 32,561) [60], which contains demographic data from the 1994 US census. Some examples, where we used different categorical attributes from the data set as *S* and *U*, are depicted in Figure 2. We omitted PolyOpt from the larger two experiments, as the space complexity became unfeasible: for *occupation* vs. *education*, the polyhedron $\hat{\Gamma }$ was 240-dimensional and was defined by 57,840 inequality constraints; to find its set of vertices Matlab needed to operate on a 57,840 × 57,840 matrix, whose size (24.7 GB) exceeded Matlab’s maximum array size.

We can see that PolyOpt clearly outperformed IR and SRR in the first two experiments, especially in the high-privacy regime (low ε). Similarly, IR outperformed SRR in the high-privacy regime, but was slightly overtaken for high ε. This is interesting, since SRR satisfies a stronger privacy guarantee, as it provides privacy for all adversary assumptions, so we expected it to offer less utility than IR. An explanation for this is that IR is forced to transmit *S* and *U* separately, and so it can be less efficient than SRR, which does not have this restriction. At any rate, the difference between IR and SRR in the low-privacy regime was only marginal compared to the advantage of PolyOpt over both. In the second two experiments, where PolyOpt was infeasible, we can see that IR clearly outperformed SRR. Overall, we see that, especially in the low-privacy regime, PolyOpt was the preferable RLDP mechanism, followed by IR and SRR. Furthermore, we can see that, in all experiments, GRR performed the worst, and the best RLDP mechanism significantly outperformed GRR. This shows that adopting RLDP as a privacy metric results in significantly better utility over LDP. Conversely, NR outperfored the RLDP methods, although the difference between NR and PolyOpt was marginal for higher ε. As for PolyOpt, NR was computationally out of reach for larger |X|.

### 8.2. Synthetic Data

**Figure 3:**
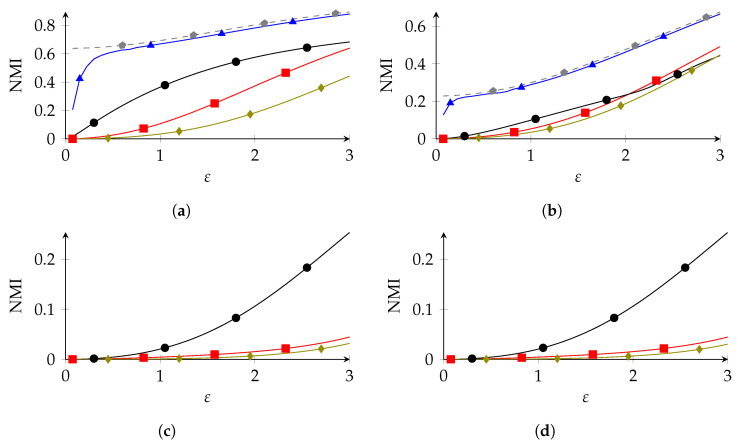
Synthetic experiments with n=32561 and β=0.05 (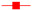 SRR, 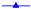 PolyOpt, 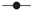 IR, 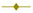 GRR, 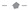 NR). (**a**) $a_{1}=2,a_{2}=5$, (**b**) $a_{1}=5,a_{2}=2$, (**c**) $a_{1}=15,a_{2}=16$, (**d**) $a_{1}=42$, $a_{2}=6$.

To study the robustness of our method with respect to utility (Section 8.4) and privacy (Section 8.3), we also needed experiments in which $P^{*}$ was known. For this, we considered experiments on synthetic data. For this, we first randomly created a probability distribution $P^{*}$ on X, where X was the same as in the experiments on the adult data set. The distribution $P^{*}$ was drawn from the Jeffreys prior on $P_{X}$, i.e., the symmetric Dirichlet distribution with parameter $\frac{1}{2}$. From $P^{*}$, we then drew n=32,561 elements of X, which we used to obtain the estimate $\hat{P}$; this estimate was then used to create the privacy mechanisms. We carried this out 100 times, and we averaged the NMI of these 100 distributions. The results are shown in Figure 3. The results were similar to those of the experiments of the adult data set: PolyOpt outperformed IR, which outperformed SRR, for small |X| SRR could overtake IR in the low-privacy regime. Furthermore, GRR was the worst overall, while NR was the best overall, but only by a small margin.

### 8.3. Realized Privacy Parameter

In the previous subsections, we saw that NR had a (marginally) better utility than PolyOpt. However, this is not a completely fair comparison, since NR was only designed to give privacy for $X∼\hat{P}$ and might result in a larger privacy leakage for $X∼P^{*}$. For the synthetic data, $P^{*}$ was known, and we could measure the true privacy leakage. For a protocol Q, we defined the *realized privacy parameter* $\varepsilon^{*}$ as

$$\begin{array}{cc} \varepsilon^{*} & =\max_{\begin{array}{c} y\in Y,\\ s_{1},s_{2}\in S \end{array}}\frac{P_{X∼P^{*}}\left(Y=y|S=s_{1}\right)}{P_{X∼P^{*}}\left(Y=y|S=s_{2}\right)}\\ \; & =\max_{\begin{array}{c} y\in Y,\\ s_{1},s_{2}\in S \end{array}}\frac{\sum_{u}Q_{y|s_{1},u}P_{u|s_{1}}^{*}}{\sum_{u}Q_{y|s_{2},u}P_{u|s_{2}}^{*}}. \end{array}$$

**Figure 4:**
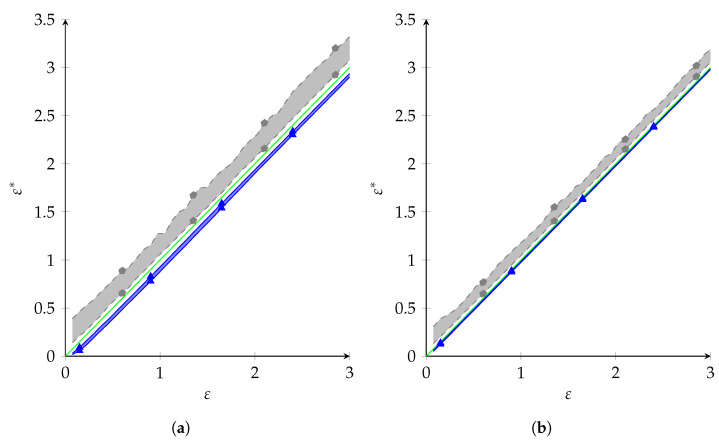
Realized privacy parameter $\varepsilon^{*}$ on synthetic data. Shaded area is bounded by the 25% and 75% quantiles (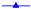 Polyopt, 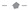 NR). The green line depicts $\varepsilon =\varepsilon^{*}$. (**a**) $a_{1}=2,a_{2}=5$, (**b**) $a_{1}=5,a_{2}=2$.

Note that this becomes *∞* when there exist s,y such that $P_{X∼P^{*}}\left(Y=y|S=s\right)=0$. We compared $\varepsilon^{*}$ for NR and PolyOpt: the results are shown in Figure 4, where we give the 25% and 75% quantiles for both protocols, out of 100 considered distributions. As one can see, NR’s $\varepsilon^{*}$ was consistently greater than ε, while PolyOpt’s $\varepsilon^{*}$ was consistently lesser. This is what we expected, as NR does not give privacy guarantees for $P^{*}$, but PolyOpt does when $P^{*}\in F$, which happens with 95% probability. Note that the privacy leakage was especially bad for low ε: at ε=0.075, the lowest value of ε we tested, the 75%-quantile of $\varepsilon^{*}$ of NR was 0.3897, which is more than 5 times the desired privacy parameter. Overall, we can conclude that NR gave marginally better utility, but this came at quite a privacy cost.

### 8.4. Utility Robustness

**Figure 5:**
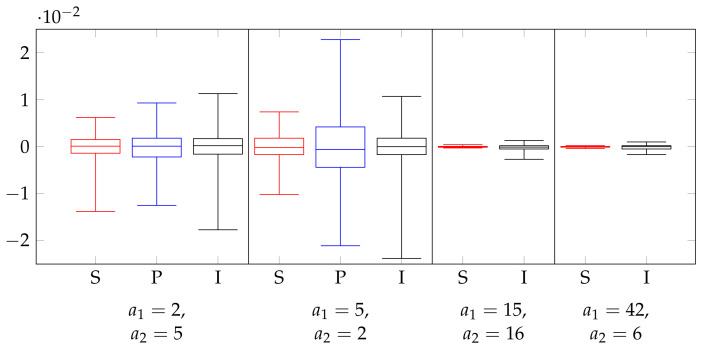
Normalized difference in NMI for $\hat{P}$ and $P^{*}$ on synthetic data (ε=1.5), measured over 100 runs. Box denotes 25–75%-quantiles, whiskers denote minima and maxima. S = SRR, P = PolyOpt, I = IR.

For the synthetic data sets (where we knew $P^{*}$), we also investigated the normalized difference in mutual information $\frac{I_{\hat{P}}\left(X;Y\right)-I_{P^{*}}\left(X;Y\right)}{I_{\hat{P}}\left(X;Y\right)}$, to see to what extent we could use $I_{\hat{P}}\left(X;Y\right)$ as a utility metric in lieu of the true utility $I_{P^{*}}\left(X;Y\right)$. This is shown for the three methods in Figure 5, at ε=1.5. Overall, we can see that the difference was quite minor: for all three methods, the difference in NMI, even at its most extreme, was less than 3% of the NMI value. Furthermore, the differences were very symmetric, with the difference being positive and negative approximately equally often. We can conclude that we were justified in using $I_{\hat{P}}\left(X;Y\right)$ as a utility metric in the other experiments.

### 8.5. Impact of β

We also considered the impact of β on utility for synthetic data (fixing ε=1.5). The results are shown in Table 2, which are averages over 100 runs. Note that SRR does not depend on β, since it assumes $F=P_{X}$. Interestingly, we can see that the impact of β was quite limited; changing β by a factor 100 had at most about 4% impact on NMI. This impact was less for PolyOpt than for IR, and less for larger X. Overall, we can conclude that by choosing β closer to 0, we can significantly increase the robustness of privacy without making a considerable impact on utility.

## 9. Conclusions and Future Work

In this paper, we presented a number of algorithms that, given a desired privacy level ε, an estimated distribution $\hat{P}$, and a bound on the Rényi divergence $D_{\alpha }(\hat{P}\left||P\right)$, return privacy mechanisms that satisfy a differential privacy-like privacy constraint for the part of the data that is considered sensitive, for all distributions *P* within the divergence bound. The first class of privacy mechanisms, PolyOpt, offers high utility, but is computationally complex, as it relies on vertex enumeration. The second class, SRR, satisfies a stronger privacy requirement and is optimal in the low-privacy regime with reference to this requirement, but as a result has less utility than mechanisms that do not satisfy this stronger privacy requirement. The third class, IR, is a general framework for releasing the sensitive and non-sensitive part of the data independently, and the optimal division of the privacy budget between these can be found via 1-dimensional optimization; thus, the optimal IR mechanism can be found quickly, while still offering decent utility. Furthermore, taking RLDP rather than LDP as a privacy constraint, i.e., protecting only the part of the data that is sensitive, significantly improves utility. In particular, we showed that the utility of PolyOpt is close to the utility of the optimal non-robust privacy mechanism. In other words, asking for robustness in privacy comes at only a small performance penalty in utility. At the same time, we showed that *not* asking for robustness comes at a substantial privacy cost.

There are various interesting directions for future research to build upon the results in this paper. One direction is to find analytical bounds on the performance gap between PolyOpt and optimal mechanisms, in particular on the gap with reference to either the non-robust optimal mechanism from [3] or with reference to an optimal robust mechanism. Note, however, that for the moment we do not have any results on optimal robust mechanisms. Another direction is to improve the performance of the low-complexity algorithms that have been proposed. For instance, in independent reporting, one could change the underlying LDP mechanism from GRR to an optimal mechanism. Since GRR is only optimal in the high-privacy regime, we expect that there would be room for improvement in the low-privacy regime. A significant challenge is incorporating optimal mechanisms along the lines of [5]; however, these mechanisms depend on $P^{*}$ which is inaccessible in the RLDP framework. Yet another interesting direction would be to incorporate robustness in utility in addition to robustness in privacy. This would require finding a mechanism that maximizes $\min_{P\in F}I_{P}\left(X;Y\right)$. The challenge in this is that $I_{P}\left(X;Y\right)$ is concave in *P*, which makes minimizing it over F difficult. Finally, it would be interesting to apply the RLDP framework to other models. In this work, we studied the model where *X* splits into a sensitive part *S* and a non-sensitive part *U*. It would be interesting to also study the more general case where *X* is correlated with the sensitive data *S*, or to apply RLDP to the models that are studied in [12].

## Figures and Tables

**Table 1:** Notation used in this paper. ‘Page’ denotes the page the notation is first defined.

Notation	Meaning	Page
S	sensitive data space	6
U	non-sensitive data space	6
X	S×U	6
$a_{1},a_{2},a,b$	|S|,|U|,|X|,|Y|	6
X=(S,U)	user data	6
ine Q	privacy mechanism	6
*Q*	matrix of Q	6
*Y*	Q(X)	6
Y	output space	6
$P_{X}$	space of prob. dist. on X	6
$P^{*}$	true distribution	6
$\hat{P}$	estimated distribution	7
I	mutual information	8
F	uncertainty set for *P*	6
$P_{U|s}$	condition probability vector	9
$F_{U|s}$	conditional projection of F	9
$L_{u|s}\left(F\right),{\mathrm{rad}}_{s}\left(F\right)$	statistics of F	9
$D_{\alpha }$	Rényi divergence	10
PolyOpt	PolyOpt	13
${\mathrm{SRR}}^{\varepsilon }$	Secret Randomized Response	17
${\mathrm{IR}}_{R^{1},R^{2}}$	Independent Reporting	18

**Table 2:** NMI for synthetic data for various values of β (ε=1.5).

	$a_{1}=2,a_{2}=5$			$a_{1}=5,a_{2}=2$		
β	0.1	0.01	0.001	0.1	0.01	0.001
SRR	0.231	0.231	0.231	0.126	0.126	0.126
PolyOpt	0.727	0.723	0.719	0.374	0.372	0.370
IR	0.512	0.501	0.492	0.169	0.165	0.162
	$a_{1}=15,a_{2}=16$			$a_{1}=42,a_{2}=6$		
β	0.1	0.01	0.001	0.1	0.01	0.001
SRR	0.009	0.009	0.009	0.005	0.005	0.005
IR	0.055	0.053	0.051	0.052	0.052	0.052

## Data Availability

Data are contained within the article.

## Appendix A. Proofs

### Appendix A.1. Proof of Theorem 1

This follows from the following four lemmas, where the RHS of (24) is denoted ${\bar{F}}_{U|s}$:

**Lemma A1.** 
*If α≠1, then $F_{U|s}\subset {\bar{F}}_{U|s}$.*

**Proof.** Assume α<1; the case α>1 is handled analogously. Then, we rewrite $D_{\alpha }(\hat{P}\left||P\right)\leq B$ as

(A1) $$\sum_{x}\frac{{\hat{P}}_{x}^{\alpha }}{P_{x}^{\alpha -1}}\geq e^{B(\alpha -1)}.$$

Let $C=e^{B(\alpha -1)}$. Then,

(A2)P^sαPsα−1∑uP^u|sαPu|sα−1=∑uP^s,uαPs,uα−1(A3)≥C−∑s′≠s∑uP^s′,uαPs′,uα−1.

For $s^{\prime }\in S\setminus \left\{s\right\}$ and u∈U, define $P_{s^{\prime },u|\neg s}=\frac{P_{u,s^{\prime }}}{1-P_{s}}$ and ${\hat{P}}_{s^{\prime },u|\neg s}=\frac{{\hat{P}}_{u,s^{\prime }}}{1-{\hat{P}}_{s}}$. Then, (A3) can be written as

(A4) $$\frac{{\hat{P}}_{s}^{\alpha }}{P_{s}^{\alpha -1}}\sum_{u}\frac{{\hat{P}}_{u|s}^{\alpha }}{P_{u|s}^{\alpha -1}}\geq C-\frac{{\left(1-{\hat{P}}_{s}\right)}^{\alpha }}{{\left(1-P_{s}\right)}^{\alpha -1}}\sum_{s^{\prime }\neq s}\sum_{u}\frac{{\hat{P}}_{s^{\prime },u|\neg s}^{\alpha }}{P_{s^{\prime },u|\neg s}^{\alpha -1}}.$$

Furthermore, $P_{•|\neg s}={\left(P_{s^{\prime },u|\neg s}\right)}_{s^{\prime }\in S\setminus \left\{s\right\},u\in U}$ and ${\hat{P}}_{•|\neg s}={\left({\hat{P}}_{s^{\prime },u|\neg s}\right)}_{s^{\prime }\in S\setminus \left\{s\right\},u\in U}$ form probability distributions on (S∖{s})×U. As such, we have

(A5) $$\sum_{u}\frac{{\hat{P}}_{s^{\prime },u|\neg s}^{\alpha }}{P_{s^{\prime },u|\neg s}^{\alpha -1}}=e^{\left(\alpha -1\right)D_{\alpha }({\hat{P}}_{•|\neg s}\left|\right|P_{•|\neg s})}\leq 1.$$

Applying this to (A4), we obtain

(A6) $$\frac{{\hat{P}}_{s}^{\alpha }}{P_{s}^{\alpha -1}}\sum_{u}\frac{{\hat{P}}_{u|s}^{\alpha }}{P_{u|s}^{\alpha -1}}\geq C-\frac{{\left(1-{\hat{P}}_{s}\right)}^{\alpha }}{{\left(1-P_{s}\right)}^{\alpha -1}}$$

or

(A7) $$\sum_{u}\frac{{\hat{P}}_{u|s}^{\alpha }}{P_{u|s}^{\alpha -1}}\geq \frac{P_{s}^{\alpha -1}}{{\hat{P}}_{s}^{\alpha }}\left(C-\frac{{\left(1-{\hat{P}}_{s}\right)}^{\alpha }}{{\left(1-P_{s}\right)}^{\alpha -1}}\right).$$

To find the bound on $\sum_{u}\frac{{\hat{P}}_{u|s}^{\alpha }}{P_{u|s}^{\alpha -1}}$, we have to minimize the RHS of this inequality. The only unknown on the right is $P_{s}$. We find the minimum value of the right-hand side by differentiating with respect to $P_{s}$, for which we obtain

(A8) $$\left(\alpha -1\right)\frac{P_{s}^{\alpha -2}{\left(1-{\hat{P}}_{s}\right)}^{\alpha }}{{\hat{P}}_{s}^{\alpha }}\left(\frac{C}{{\left(1-{\hat{P}}_{s}\right)}^{\alpha }}-\frac{1}{{\left(1-P_{s}\right)}^{\alpha }}\right).$$

Setting this equal to 0, we find $P_{s}=1-C^{-1/\alpha }\left(1-{\hat{P}}_{s}\right)$. Substituting this into (A7), we obtain

(A9) $$\sum_{u}\frac{{\hat{P}}_{u|s}^{\alpha }}{P_{u|s}^{\alpha -1}}\geq \frac{{\left(C^{1/\alpha }-\left(1-{\hat{P}}_{s}\right)\right)}^{\alpha }}{{\hat{P}}_{s}^{\alpha }}$$

which can be written as

(A10) $$D_{\alpha }({\hat{P}}_{U|s}\left|\right|P_{U|s})\leq \frac{\alpha }{\alpha -1}\log\left(\frac{e^{(\alpha -1)B/\alpha }-\left(1-{\hat{P}}_{s}\right)}{{\hat{P}}_{s}}\right),$$

showing that $P_{U|s}\in {\bar{F}}_{U|s}$. Since *P* was chosen arbitrarily, we can conclude $F_{U|s}\subset {\bar{F}}_{U|s}$. □

**Lemma A2.** 
*If α≠1, then ${\bar{F}}_{U|s}\subset F_{U|s}$.*

**Proof.** Again we assume α<1. Suppose that $R\in P_{U}$ satisfies $D_{\alpha }({\hat{P}}_{U|s}\left||R\right)\leq B_{s}$. Let *C* be as in (A1) and define $\gamma =1-C^{-1/\alpha }\left(1-{\hat{P}}_{s}\right)$; then,

(A11)1α−1log∑uP^u|sαRuα−1=Dα(P^U|s||R)(A12)≤Bs(A13)=αα−1loge(α−1)B/α−(1−P^s)P^s(A14)=αα−1logC1/αγP^s

which we can express as

(A15) $$\sum_{u}\frac{{\hat{P}}_{u|s}^{\alpha }}{R_{u}^{\alpha -1}}\geq \frac{C\gamma^{\alpha }}{{\hat{P}}_{s}^{\alpha }}.$$

Define $P\in P_{X}$ by

(A16) $$P_{u,s^{\prime }}=\left\{\begin{array}{cc} \gamma R_{u}, & \mathrm{if}\;s^{\prime }=s,\\ C^{-1/\alpha }{\hat{P}}_{u,s^{\prime }} & \mathrm{otherwise}. \end{array}\right.$$

Then, $P_{U|s}=R$, and

(A17)∑u,s′P^u,s′αPu,s′α−1=∑uP^u,sαγα−1Ruα−1+∑u∑s′≠sC1/αP^u,s′(A18)=P^sαγα−1∑uP^u|sαRuα−1+C(α−1)/α(1−P^s)(A19)≥γC+C(α−1)/α(1−P^s)(A20)=C.

As in the proof of Lemma A1, the condition $\sum_{u,s^{\prime }}\frac{{\hat{P}}_{u,s^{\prime }}^{\alpha }}{P_{u,s^{\prime }}^{\alpha -1}}\geq C$ is equivalent to $D_{\alpha }(\hat{P}\left||P\right)\leq B$. Thus, we can conclude that P∈F and so $R=P_{U|s}\in F_{U|s}$. Since *R* was chosen arbitrary, this shows ${\bar{F}}_{U|s}\subset F_{U|s}$. □

**Lemma A3.** 
*If α=1, then $F_{U|s}\subset {\bar{F}}_{U|s}$.*

**Proof.** Let P∈F, and define $P_{•|\neg s},{\hat{P}}_{•|\neg s}$ as in the proof of Lemma A1. Then,

(A21)D1(P^||P)=P^s∑uP^u|slogP^sP^u|sPsPu|s+(1−P^s)∑s′,uP^u,s′|¬s(1−P^s)P^u,s′|¬s(1−Ps)Pu,s′|¬s(A22)=P^sD1(P^U|s||PU|s)+(1−P^s)D1(P^•|¬s||P•|¬s)+P^slogP^sPs+(1−P^s)log1−P^s1−Ps(A23)=P^sD1(P^U|s||PU|s)+(1−P^s)D1(P^•|¬s||P•|¬s)+D1(VP^s||VPs),

where for p∈[0,1], the random variable $V_{p}$ is defined to follow a Bernoulli distribution with $P(V_{p}=1)=p$. Since $D_{1}$ is non-negative and $D_{1}(\hat{P}\left||P\right)\leq B$, we find

(A24)D1(P^U|s||PU|s)=1P^sD1(P^||P)−(1−P^s)D1(P^•|¬s||P•|¬s)−D1(VP^s||VPs)(A25)≤D1(P^||P)P^s(A26)≤BP^s.

Thus, $P_{U|s}\in {\bar{F}}_{U|s}$; since P∈F was chosen arbitrary, we can conclude $F_{U|s}\subset {\bar{F}}_{U|s}$. □

**Lemma A4.** 
*If α=1, then ${\bar{F}}_{U|s}\subset F_{U|s}$.*

**Proof.** Let $R\in P_{U}$ be such that $D_{1}({\hat{P}}_{U|s}\left||R\right)\leq \frac{B}{{\hat{P}}_{s}}$. Define $P\in P_{X}$ by

(A27) $$P_{u,s^{\prime }}=\left\{\begin{array}{cc} {\hat{P}}_{s}R_{u}, & \;\mathrm{if}\;s=s^{\prime },\\ {\hat{P}}_{u,s^{\prime }} & \;\mathrm{if}\;s\neq s^{\prime }. \end{array}\right.$$

Then $P_{U|s}=R$. Furthermore, in (A23) one has $D_{1}({\hat{P}}_{•|\neg s}\left|\right|P_{•|\neg s})=D_{1}(V_{{\hat{P}}_{s}}\left|\right|V_{P_{s}})=0$, and so $D_{1}(\hat{P}\left||P\right)\leq B$. This shows that P∈F, and so $R=P_{U|s}\in F_{U|s}$. Since *R* was chosen arbitrarily, we can conclude that ${\bar{F}}_{U|s}\subset F_{U|s}$. □

### Appendix A.2. Proof of Proposition 1

We first prove the following two auxiliary lemmas. We only prove these for α>1; the other cases are handled analogously.

**Lemma A5.** 
*Let x∈X, and define*

(A28)ξ−(ρ)=infξ∈(0,1]:EB(ρ,ξ)≤0,(A29)ξ+(ρ)=supξ∈[1,(1−ρ)−1):EB(ρ,ξ)≤0,

*where $E_{B}$ is as in Proposition 1. Then, $\min_{P\in F}P_{x}={\hat{P}}_{x}\xi_{-}\left({\hat{P}}_{x}\right)$ and $\max_{P\in F}={\hat{P}}_{x}\xi_{+}\left({\hat{P}}_{x}\right)$.*

**Proof.** As in the proof of Lemma A1, define $C=e^{(\alpha -1)B}$; thus

(A30) $$F=\left\{P\in P_{X}:\sum_{x^{\prime }\in X}\frac{{\hat{P}}_{x^{\prime }}^{\alpha }}{P_{x^{\prime }}^{\alpha -1}}\leq C\right\}.$$

Furthermore, define a function *F* by

(A31) $$F\left(\rho ,\xi \right)=\rho \xi^{1-\alpha }+\left(1-\rho \right){\left(\frac{1-\rho \xi }{1-\rho }\right)}^{1-\alpha }-C,$$

with $F\left(1,\xi \right)=\xi^{1-\alpha }-C$ the limit as ρ→1. Then, $E_{B}\left(\rho ,\xi \right)=\frac{1}{\alpha -1}\log\left(F\left(\rho ,\xi \right)+e^{(\alpha -1)B}\right)-B$, so $F\left(\rho ,\xi \right)\leq 0\Leftrightarrow E_{B}\left(\rho ,\xi \right)\leq 0$. Thus, $\xi_{-}\left(\rho \right)=\inf\left\{\xi \in \left[0,1\right]:F\left(\rho ,\xi \right)\leq 0\right\}$ and the analogous statement holds for $\xi_{+}\left(\rho \right)$. The *P* that yield the extremal $P_{x}$ lie on the boundary of F; hence, they either satisfy $P_{x}\in \left\{0,1\right\}$, or the equality

(A32) $$\sum_{x^{\prime }\in X}\frac{{\hat{P}}_{x^{\prime }}^{\alpha }}{P_{x^{\prime }}^{\alpha -1}}=C.$$

In the latter case, the extremal values of $P_{x}$ have to be stationary points of the Lagrangian expression

(A33) $$P_{x}+\lambda \left(\sum_{x^{\prime }}\frac{{\hat{P}}_{x^{\prime }}^{\alpha }}{P_{x^{\prime }}^{\alpha -1}}-C\right)+\mu \left(\sum_{x^{\prime }}P_{x^{\prime }}-1\right)=0.$$

Taking derivatives with respect to all $P_{x}$, we find

(A34)1+(1−α)λP^xαPxα+μ=0,(A35)∀x′≠x:(1−α)λP^x′αPx′α+μ=0.

It follows that $P_{x}={\left(\frac{(\alpha -1)\lambda }{\mu -1}\right)}^{1/\alpha }{\hat{P}}_{x}=:\xi {\hat{P}}_{x}$ and $P_{x^{\prime }}={\left(\frac{(\alpha -1)\lambda }{\mu }\right)}^{1/\alpha }{\hat{P}}_{x^{\prime }}=:\psi {\hat{P}}_{x^{\prime }}$ for all $x^{\prime }\neq x$, where ξ and ψ do not depend on *x* or $x^{\prime }$. We can find $\xi ,\psi \in R_{\geq 0}$ by solving the joint set of equations

(A36)C=∑x′P^x′αPx′α−1(A37)=P^xαPxα−1+∑x′≠xP^x′αPx′α−1(A38)=P^xξ1−α+(1−P^x)ψ1−α,(A39)1=∑x′Px′(A40)=P^xξ+(1−P^x)ψ.

Define $\rho ={\hat{P}}_{x}$. Then, (A40) implies $\psi =\frac{1-\rho \xi }{1-\rho }$, and the condition ψ≥0 is equivalent to $\xi \leq \rho^{-1}$. Substituting this into (A38) shows that we find ξ by solving F(ρ,ξ)=0 for $\xi \in (0,{\left(1-\rho \right)}^{-1})$. Since F(ρ,1)=1−C<0 and *F* is strictly convex in ξ, there exists, at most, one solution in (0,1] and, at most, one in $\left[1,{\left(1-\rho \right)}^{-1}\right)$. It follows that (A33) has, at most, two stationary points, which must correspond to the minimal and maximal value of $P_{x}$. If the solution in (0,1] exists, it is equal to $\xi_{-}\left(\rho \right)$, and this stationary point of (A33) corresponds to the minimal value of $P_{x}$, which is then equal to ${\hat{P}}_{x}\xi_{-}\left({\hat{P}}_{x}\right)$. If the solution in (0,1] does not exist, then the minimal value of $P_{x}$ is not attained on the boundary and is equal to 0, which then is also equal to ${\hat{P}}_{x}\xi_{-}\left({\hat{P}}_{x}\right)$. Either way, we find

(A41) $$\min_{P\in F}P_{x}={\hat{P}}_{x}\xi_{-}\left({\hat{P}}_{x}\right).$$

The proof for the maximal value of $P_{x}$ is analogous. □

**Lemma A6.** 
*For $X_{1}\subset X$ define ${\hat{P}}_{X_{1}}:=\sum_{x\in X_{1}}{\hat{P}}_{x}$. Then,*

(A42)
$$\underset{P\in F}{\sup}||P-\hat{P}{\left|\right|}_{1}=2\max_{\begin{array}{c} X_{1}\subset X:\\ X_{1}\neq ⌀ \end{array}}{\hat{P}}_{X_{1}}\left(\xi_{+}\left({\hat{P}}_{X_{1}}\right)-1\right).$$

**Proof.** For a given *P*, define $X_{1}=\left\{x\in X:P_{x}\geq {\hat{P}}_{x}\right\}$ and $X_{2}=\left\{x\in X:P_{x}<{\hat{P}}_{x}\right\}$. To find the maximal value of $||P-\hat{P}{\left|\right|}_{1}$, we first maximize it for a given partition $X_{1},X_{2}$ of X, and then we maximize over all partitions. Note that $X_{1}=⌀$ is impossible, and for $X_{1}=X$, we have $P=\hat{P}$, which is certainly not optimal. Given $X_{1},X_{2}$, one has

(A43) $$||P-\hat{P}{\left|\right|}_{1}=\sum_{x\in X_{1}}\left(P_{x}-{\hat{P}}_{x}\right)+\sum_{x\in X_{2}}\left({\hat{P}}_{x}-P_{x}\right).$$

As before, the *P* maximizing this lies either on the boundary of the probability simplex or it satisfies (A32). For the latter case, we have the Lagrangian expression

(A44) $$\sum_{x\in X_{1}}\left(P_{x}-{\hat{P}}_{x}\right)+\sum_{x\in X_{2}}\left({\hat{P}}_{x}-P_{x}\right)+\lambda \left(\sum_{x}\frac{{\hat{P}}_{x}^{\alpha }}{P_{x}^{\alpha -1}}-C\right)+\mu \left(\sum_{x}P_{x}-1\right)=0.$$

Taking derivatives, we find, analogously to (A34)–(A35), that there exist ξ,ψ such that $P_{x}=\xi {\hat{P}}_{x}$ for all $x\in X_{1}$ and $P_{x}=\psi {\hat{P}}_{x}$ for all $x\in X_{2}$. By definition of $X_{1}$ and $X_{2}$, we have ξ≥1 and 0≤ψ<1. Analogously to (A36)–(A40), these have to satisfy

(A45)P^X1ξ1−α+(1−P^X1)ψ1−α=C,(A46)P^X1ξ+(1−P^X1)ψ=1.

From this point onward, this proof is analogous to that of Lemma A5. Let $\rho ={\hat{P}}_{X_{1}}$. Expressing ψ in terms of ξ and substituting this means that to find ξ we have to solve F(ρ,ξ)=0 for $\xi \in [1,{\left(1-\rho \right)}^{-1})$, where *F* is as in the proof of Lemma A5. As before, at most, one such solution exists, and when it does, it corresponds to the maximal value of $||P-\hat{P}{\left|\right|}_{1}$ (given $X_{1}$). If it does not exist, then the maximal value of $||P-\hat{P}{\left|\right|}_{1}$ is obtained at the boundary where ${\hat{P}}_{X_{1}}=1$. Either way the maximum is obtained when $\xi =\xi_{+}\left(\rho \right)$, which means that

(A47)||P−P^||1=∑x∈X1(Px−P^x)+∑x∈X2(P^x−Px)(A48)=∑x∈X1P^x(ξ+(ρ)−1)+∑x∈X2P^x1−1−ρξ+(ρ)1−ρ(A49)=ρ(ξ+(ρ)−1)+(1−ρ)1−1−ρξ+(ρ)1−ρ(A50)=2ρ(ξ+(ρ)−1).

This is the maximal value of $||P-\hat{P}{\left|\right|}_{1}$ given $X_{1}$; we now find the overall maximum by maximizing over all non-empty $X_{1}$. □

**Proof** **of** **Proposition** **1.** In Lemmas A5 and A6, take U instead of X, ${\hat{P}}_{U|s}$ instead of $\hat{P}$, and $B_{s}$ instead of *B*. Then, by Theorem 1, the role of F is taken by $F_{U|s}$. Thus, applying Lemmas A5 and A6 gives us Proposition 1 directly. □

### Appendix A.3. Proof of Lemma 1 and Proposition 2

As in the proof of Proposition 1, since by Theorem 1 the projected set $F_{U|s}$ is defined by a Rényi divergence as is F, it suffices to prove the analogous statements about F rather than $F_{U|s}$. Concretely, we prove the following:

**Lemma A7.** 
*Suppose α=2 and define $\tilde{B}=e^{B}-1$; let $\xi_{\pm }$ be as in Lemma A5. Then,*

(A51)
$$\begin{array}{cc} \xi_{\pm }\left(\rho \right) & =\frac{\tilde{B}+2\rho \pm \sqrt{{\tilde{B}}^{2}+4\rho \tilde{B}-4\tilde{B}\rho^{2}}}{2\rho (\tilde{B}+1)}. \end{array}$$

*Furthermore, the following hold:*

1. *Let $x_{\min}=\arg\min_{x\in X}{\hat{P}}_{x}$. If $\tilde{B}\geq {\left(1-{\hat{P}}_{x_{\min}}\right)}^{2}$, then the maximum in (A6) is attained at $X_{1}=\left\{x_{\min}\right\}$.*
2. *If $\tilde{B}<{\left(1-{\hat{P}}_{x_{\min}}\right)}^{2}$ one has $\sup_{P\in F}||P-\hat{P}{\left|\right|}_{1}\leq \sqrt{\tilde{B}}$.*

The formulas here look slightly different from those in Lemma 1 and Proposition 2. We use this form because it makes the proof more convenient: replacing $\tilde{B}$ with $e^{B}-1$ throughout yields exactly the results of Lemma 1 and Proposition 2 for F instead of $F_{U|s}$.

**Proof.** Consider the function F(ρ,ξ) from (A31) for α=2 and $C=\tilde{B}+1$, i.e.,

(A52) $$F\left(\rho ,\xi \right)=\frac{\rho }{\xi }+\frac{{\left(1-\rho \right)}^{2}}{1-\rho \xi }-\tilde{B}-1.$$

Then, F(ρ,ξ)=0 can be rewritten to a quadratic equation in ξ. Its two roots are $\xi_{\pm }\left(\rho \right)$, and with some rewriting they can be expressed as in (31). For points 1 and 2, we note that

(A53) $$2\rho \left(\xi_{+}\left(\rho \right)-1\right)=\frac{\tilde{B}-2\tilde{B}\rho +\sqrt{{\tilde{B}}^{2}+4\tilde{B}\rho -4\tilde{B}\rho^{2}}}{\tilde{B}+1}.$$

We can find its extremal values with respect to ρ by taking the derivative and setting it to 0, i.e., by solving

(A54) $$-\frac{2\tilde{B}}{\tilde{B}+1}+\frac{2\tilde{B}-4\tilde{B}\rho }{\left(\tilde{B}+1\right)\sqrt{{\tilde{B}}^{2}+4\tilde{B}\rho -4\tilde{B}\rho^{2}}}=0,$$

which has a single solution $\rho_{\mathrm{opt}}=\frac{1-\sqrt{\tilde{B}}}{2}$. Since (A53) is concave in ρ, this means that this unique extremal value is a maximum. If $\tilde{B}\geq {\left(1-2{\hat{P}}_{x_{\min}}\right)}^{2}$, then $\rho_{\mathrm{opt}}\geq {\hat{P}}_{x_{\min}}$, and $\rho (\xi_{+}\left(\rho \right)-1)$ is decreasing in ρ on $\left[{\hat{P}}_{x_{\min}},1\right]$. Since all possible values of ${\hat{P}}_{X_{1}}$ lie in this interval, it is optimal to take $X_{1}$ such that ${\hat{P}}_{X_{1}}$ is minimized, i.e., $X_{1}=\left\{x_{\min}\right\}$; this proves point 1. For point 2 we have (and also for general *B*)

(A55)supP∈F||P−P^||1=2maxX1⊂X:X1≠⌀P^X1(ξ+(P^X1)−1)(A56)≤2ρopt(ξ+(ρopt)−1)(A57)=B˜−2B˜1−B˜2+B˜2+4B˜1−B˜2−4B˜(1−B˜)24B˜+1(A58)=B˜.

□

### Appendix A.4. Proof of Theorem 2

Let $D=\prod_{s\in S}D_{s}\subset R^{X}$. Thus, an element t∈D is of the form $t={\left(t_{s,u}\right)}_{(s,u)\in X}$, and for any *s*, we have ${\left(t_{s,u}\right)}_{u\in U}\in D_{s}$. For $s_{1},s_{2}\in S$, let $B^{s_{1},s_{2}}\in R^{X\times X}$ be the matrix given by

(A59) $$B_{\left(s,u\right);\left(s^{\prime },u^{\prime }\right)}^{s_{1},s_{2}}=\left\{\begin{array}{cc} 1, & \mathrm{if}\;u=u^{\prime }\mathrm{and}s=s^{\prime }=s_{1},\\ -e^{\varepsilon }, & \mathrm{if}\;u=u^{\prime }\mathrm{and}s=s^{\prime }=s_{2},\\ 0, & \mathrm{otherwise}. \end{array}\right.$$

Then, we can rewrite (41) as

(A60) $$\forall y,s_{1},s_{2}:\max_{t\in D}{\left({\left(B^{s_{1},s_{2}}\right)}^{T}Q_{y}\right)}^{T}t\leq 0.$$

Recall that for each *s*, we have $D_{s}=\left\{R\in P_{U}:R_{u}\geq L_{u|s}\right\}$. Since $D=\prod_{s}D_{s}$, we can write

(A61)D=t∈RX:∀s,u:ts,u≥Lu|s,∀s:∑uts,u=1(A62)={t∈RX:Φt+ϕ≥0,Ψt+ψ=0},

where $\Phi \in R^{X\times X}$, $\phi \in R^{X}$, $\Psi \in R^{S\times X}$ and $\psi \in R^{S}$ are given, for $s,s^{\prime }\in S$ and u∈U, by

(A63)Φ=idX,(A64)ϕs,u=−Lu|s,(A65)Ψs′;(s,u)=1,if s=s′,0,otherwise,(A66)ψs=−1.

Combining this with (A60), we find that Q satisfies (ε,F)-RLDP whenever

(A67) $$\forall y,s_{1},s_{2}:\max_{\begin{array}{c} t\in R^{X}:\\ \Phi t+\phi \geq 0,\\ \Psi t+\psi =0 \end{array}}{\left({\left(B^{s_{1},s_{2}}\right)}^{T}Q_{y}\right)}^{T}t\leq 0.$$

Now fix $y,s_{1},s_{2}$, and consider the linear programming problem that forms the LHS of (A67). From the duality of linear programming, we know

(A68) $$\max_{\begin{array}{c} t\in R^{X}:\\ \Phi t+\phi \geq 0,\\ \Psi t+\psi =0 \end{array}}{\left({\left(B^{s_{1},s_{2}}\right)}^{T}Q_{y}\right)}^{T}t=\min_{\begin{array}{c} z\in R^{X},w\in R^{S}:\\ \Phi^{T}z+\Psi^{T}w=-{\left(B^{s_{1},s_{2}}\right)}^{T}Q_{y},\\ z\geq 0 \end{array}}\phi^{T}z+\psi^{T}w.$$

We focus on the linear programming problem of the RHS. The terms of this problem are given by

(A69)ΦTz=z,(A70)(ΨTw)s,u=ws,(A71)((Bs1,s2)TQy)s,u=Qy|s1,u,if s=s1,−eεQy|s2,u,if s=s2,0,otherwise,(A72)ϕTz=−∑s,uLu|szs,u,(A73)ψTw=−∑sws.

The equation $\Phi^{T}z+\Psi^{T}w=-{\left(B^{s_{1},s_{2}}\right)}^{T}Q_{y}$ can now be rewritten as

(A74) $$z_{s,u}=\left\{\begin{array}{cc} -Q_{y|s_{1},u}-w_{s_{1}}, & \mathrm{if}\;s=s_{1},\\ e^{\varepsilon }Q_{y|s_{2},u}-w_{s_{2}}, & \mathrm{if}\;s=s_{2},\\ -w_{s} & \mathrm{otherwise}. \end{array}\right.$$

Thus, the restriction z≥0 translates to

$$\begin{array}{cc} w_{s_{1}} & \leq -\max_{u\in U}Q_{y|s_{1},u},\\ w_{s_{2}} & \leq e^{\varepsilon }\min_{u\in U}Q_{y|s_{2},u},\\ \forall s\neq s_{1},s_{2}:w_{s} & \leq 0. \end{array}$$

Furthermore, the objective function $\phi^{T}z+\psi^{T}w$ becomes

(A75) $$-\sum_{s}\left(1-\sum_{u}L_{u|s}\right)w_{s}+\sum_{u}Q_{y|s_{1},u}L_{u|s_{1}}-e^{\varepsilon }\sum_{u}Q_{y|s_{2},u}L_{u|s_{2}}.$$

Combining this with (A67) and (A68), we see that a sufficient condition for Q to be (ε,F)-RLDP is if there exists a $w\in R^{S}$ such that

(A76)−∑s1−∑uLu|sws+∑uQy|s1,uLu|s1−eε∑uQy|s2,uLu|s2≤0,(A77)ws1≤−maxu∈UQy|s1,u,(A78)ws2≤eεminu∈UQy|s2,u,(A79)∀s≠s1,s2:ws≤0.

Since $\sum_{u}L_{u|s}\leq 1$ for all *s*, it follows that the left-hand side of (A76) is minimal if each $w_{s}$ attains its maximal value, subject to the constraints (A77)–(A79). Substituting this, we find that the minimum of the left-hand side is equal to

(A80)1−∑uLu|s1maxu1Qy|u1,s1−eε1−∑uLu|s2minu2Qy|u2,s2+∑uQy|s1,uLu|s1−eε∑uQy|s2,uLu|s2(A81)=maxu1,u2∈U1−∑uLu|s1Qy|u1,s1−eε1−∑uLu|s2Qy|u2,s2+∑uQy|s1,uLu|s1−eε∑uQy|s2,uLu|s2(A82)=maxu1,u2∈UQy|u1,s1−eεQy|u2,s2+∑uLu|s1(Qy|s1,u−Qy|s1,u1)−eε∑uLu|s2(Qy|s2,u−Qy|s2,u2).

This has to be nonpositive for all choices of $u_{1},u_{2},s_{1},s_{2},y$; but this is true precisely if $Q_{y}\in \Gamma_{L,\varepsilon }$ for all *y*.

### Appendix A.5. Proof of Theorem 3

This is essentially analogous to the proof of Theorem 4 in [5]; the main difference is that the equivalent of $\hat{\Gamma }$ is a hypercube, for which a vertex enumeration step is not needed. Let Q be a mechanism such that $Q_{y}\in \Gamma$ for all *y*; then there exist $\alpha_{y}\in R_{\geq 0}$, $\gamma_{y}\in \hat{\Gamma }$ such that $Q_{y}=\alpha_{y}\gamma_{y}$. One has

(A83) $$I_{\hat{P}}\left(X;Y\right)=\sum_{y}\mu \left(Q_{y}\right)=\sum_{y}\alpha_{y}\mu \left(\gamma_{y}\right).$$

Since $\hat{\Gamma }$ is the convex hull of V, we can write $\gamma_{y}=\sum_{v}\lambda_{y,v}v$ for suitable constants $\lambda_{y,v}$. Define $\theta \in R_{\geq 0}^{V}$ by $\theta_{v}=\sum_{y}\lambda_{y,v}\alpha_{y}$. Then,

(A84) $$\sum_{v}\theta_{v}v=\sum_{y}Q_{y}=1_{X}.$$

As such, the matrix $Q^{\prime }\in R^{V\times X}$ defined by $Q_{v}^{\prime }=\theta_{v}v$ defines a privacy mechanism $Q^{\prime }$. One has

(A85)IP^(X;Q′(X))=∑vμ(Qv′)(A86)=∑vθvμ(v)(A87)=∑yαy∑vλy,vμ(v)(A88)≥∑yαyμ∑vλy,vv(A89)=IP^(X;Q(X)),

where we use the fact that μ is convex. This shows that the $Q_{y}$ of the optimal mechanism satisfying Theorem 2 are all of the form $\theta_{v}\cdot v$; hence, (46) yields the optimal mechanism. To see that |Y|≤a, observe that the polyhedron described in (46) is defined by *a* equality constraints, and |V| inequality constraints of the form $\theta_{v}\geq 0$. Hence, any vertex of this polyhedron has at most *a* nonzero coefficients. Since the optimal mechanism corresponds to such a vertex, and its output space Y corresponds to its nonzero coefficients, we conclude that |Y|≤a. □

### Appendix A.6. Proof of Theorem 4

We follow the proof of Theorem 14 in [5]; however, we first need the following auxiliary lemma.

**Lemma A8.** 
*Let ε>0, and let $C\subset R_{\geq 0}^{X}$ be the positive cone defined by*

(A90)
$$C=\{C\in R_{\geq 0}^{X}:C_{s,u}\leq e^{\varepsilon }C_{s^{\prime },u^{\prime }}\mathrm{for}\mathrm{all}s\neq s^{\prime }\in S,u\in U\}.$$

*Define the sets $V_{1},V_{2},V\subset R_{\geq 0}^{X}$ by*

(A91)V1=v∈R≥0X:∃s s.t.∀u:vs,u∈{e−ε,eε};∀s′≠s,∀u:vs′,u=1,(A92)V2=v∈R≥0X:∀x:vx∈{1,eε},|{s:∃u s.t.vs,u=eε}|≥2,(A93)V=V1∪V2.

*Then V spans C as a positive cone, i.e.,*

(A94)
$$C=\left\{\sum_{v\in V}\theta_{v}v:\theta \in R_{\geq 0}^{V}\right\}.$$

**Proof.** For every s∈S and $u,u^{\prime }\in U$, we have

(A95) $$C_{s,u}\leq e^{\varepsilon }C_{s^{\prime },u}\leq e^{2\varepsilon }C_{s,u^{\prime }},$$

where $s^{\prime }\in S\setminus \left\{s\right\}$ is arbitrary. Thus, in every C∈C two coefficients can differ by at most a factor $e^{\varepsilon }$ if they have different *s*, and at most a factor $e^{2\varepsilon }$ if they have the same *s*. On the extremal rays of C, the inequalities become equalities. By rescaling by a positive scalar, if necessary, we see that C is spanned by vectors of which each coefficient is in the set $\left\{e^{-\varepsilon },1,e^{\varepsilon }\right\}$. In other words, if $V^{\prime }={\left\{e^{-\varepsilon },1,e^{\varepsilon }\right\}}^{X}\cap C$, then

(A96) $$C=\mathrm{Span}(V^{\prime }),$$

where Span refers to the span as in (A94). To determine $V^{\prime }$ we consider two situations: either *v* contains both $e^{-\varepsilon }$ and $e^{\varepsilon }$ as coefficients, or not. Suppose *v* contains $e^{-\varepsilon }$ and $e^{\varepsilon }$, say $v_{s,u}=e^{-\varepsilon }$ and $v_{s^{\prime },u^{\prime }}=e^{\varepsilon }$. By (A90), we must have $s=s^{\prime }$, and by (A95), this means that $v_{s^{\prime \prime },u^{\prime \prime }}=1$ for $s^{\prime \prime }\neq s$ and any $u^{\prime \prime }$. Thus, we define, for any s∈S, the set

(A97) $$V_{s}^{\prime }=\left\{v\in R_{\geq }^{X}:\forall s^{\prime }\neq s\forall u:v_{s^{\prime },u}=1\right\}.$$

It is straightforward to show that $V_{s}^{\prime }\subset C$, and by the discussion above any $v\in V^{\prime }$ containing both $e^{-\varepsilon }$ and $e^{\varepsilon }$ is in ${⋃}_{s}V_{s}^{\prime }$. Suppose *v* does not contain both $e^{-\varepsilon }$ and $e^{\varepsilon }$, then $v\in V_{2}^{\prime }\cup V_{3}^{\prime }$ where

(A98) $$\begin{array}{cc} V_{2}^{\prime } & ={\left\{1,e^{\varepsilon }\right\}}^{X}, \end{array}$$

(A99) $$\begin{array}{cc} V_{3}^{\prime } & ={\left\{e^{-\varepsilon },1\right\}}^{X}. \end{array}$$

Furthermore, it is easy to see that $V_{2}^{\prime }\cup V_{3}^{\prime }\subset V^{\prime }$. Thus, we conclude that

(A100) $$V^{\prime }=\left(\underset{s\in S}{⋃}V_{s}^{\prime }\right)\cup V_{2}^{\prime }\cup V_{3}^{\prime }.$$

To obtain from $V^{\prime }$ to V, we throw out some vectors that are not needed to span C. We start with $V_{s}^{\prime }$. Given *s*, define the set

(A101) $$V_{s}=\left\{v\in R_{\geq 0}^{X}:\begin{array}{c} \forall u:v_{s,u}\in \left\{e^{-\varepsilon },e^{\varepsilon }\right\};\\ \forall s^{\prime }\neq s,\forall u:v_{s^{\prime },u}=1 \end{array}\right\}.$$

It is clear that $V_{s}\subset V_{s}^{\prime }$; we claim that

(A102) $$\mathrm{Span}\left(V_{s}\right)=\mathrm{Span}\left(V_{s}^{\prime }\right).$$

To see this, let $v\in V_{s}^{\prime }\setminus V_{s}$, and define $v^{-},v^{+}\in R_{\geq 0}^{X}$ by

(A103) $$\begin{array}{cc} v_{s^{\prime },u}^{+} & =\left\{\begin{array}{cc} e^{-\varepsilon }, & \mathrm{if}\;s^{\prime }=s\mathrm{and}v_{s^{\prime },u}=e^{-\varepsilon },\\ 1, & \mathrm{if}\;s^{\prime }\neq s,\\ e^{\varepsilon }, & \mathrm{if}\;s^{\prime }=s\mathrm{and}v_{s^{\prime },u}\in \left\{1,e^{\varepsilon }\right\}, \end{array}\right. \end{array}$$

(A104) $$\begin{array}{cc} v_{s^{\prime },u}^{-} & =\left\{\begin{array}{cc} e^{-\varepsilon }, & \mathrm{if}\;s^{\prime }=s\mathrm{and}v_{s^{\prime },u}\in \left\{e^{-\varepsilon },1\right\},\\ 1, & \mathrm{if}\;s^{\prime }\neq s,\\ e^{\varepsilon }, & \mathrm{if}\;s^{\prime }=s\mathrm{and}v_{s^{\prime },u}=e^{\varepsilon }. \end{array}\right. \end{array}$$

In other words, $v^{\pm }$ takes all *s*-coefficients of *v* that are equal to 1 and changes them to $e^{\pm \varepsilon }$. Then, $v^{+},v^{-}\in V_{s}^{\prime }$ and

(A105) $$v=\frac{1}{e^{\varepsilon }+1}v^{+}+\frac{e^{\varepsilon }}{e^{\varepsilon }+1}v^{-}.$$

Thus, $v\in \mathrm{Span}(V_{s}^{\prime })$, proving (A102). We now consider $V_{2}^{\prime }$ and $V_{3}^{\prime }$. First note that $V_{3}^{\prime }=e^{-\varepsilon }V_{2}^{\prime }$, so

(A106) $$\mathrm{Span}\left(V_{2}^{\prime }\right)=\mathrm{Span}\left(V_{3}^{\prime }\right).$$

We furthermore claim that

(A107) $$\mathrm{Span}\left(V_{2}\cup \underset{s\in S}{⋃}V_{s}\right)=\mathrm{Span}\left(V_{2}^{\prime }\cup \underset{s\in S}{⋃}V_{s}\right),$$

where $V_{2}$ is as in (A92). Note that clearly $V_{2}\subset V_{2}^{\prime }$. To see (A107), let $v\in V_{2}^{\prime }\setminus V_{2}$; this means that there is at most a single (s,u) such that $v_{s,u}=e^{\varepsilon }$. If no such (s,u) exists, then $v=1_{X}$, the constant vector with all ones. This implies that $e^{\varepsilon }v\in V_{2}$, showing that $v\in \mathrm{Span}(V_{2})$. Now suppose that there is exactly one (s,u) such that $v_{s,u}=e^{\varepsilon }$. Then,

(A108) $$v_{s^{\prime },u^{\prime }}=\left\{\begin{array}{cc} e^{\varepsilon }, & \mathrm{if}\;s=s^{\prime }\mathrm{and}u=u^{\prime },\\ 1, & \mathrm{otherwise}. \end{array}\right.$$

But then we can construct $v^{+}$ as in (A103) and $v^{-}$ as in (A104), and again we find

(A109) $$v=\frac{1}{e^{\varepsilon }+1}v^{+}+\frac{e^{\varepsilon }}{e^{\varepsilon }+1}v^{-}\in \mathrm{Span}\left(V_{s}\right).$$

This proves (A107). Combining (A102), (A106) and (A107) we obtain

(A110)C=SpanV2′∪V3′∪⋃s∈SVs′(A111)=SpanV2∪⋃s∈SVs(A112)=SpanV2∪V1.

□

**Proof** **of** **Theorem** **4.** We follow the proof of Theorem 14 in [5]. For $C\in R_{\geq 0}^{X}$, define

(A113) $$\mu \left(C\right)=\sum_{x}P_{x}C_{x}\log\frac{C_{x}}{\sum_{x^{\prime }}P_{x^{\prime }}C_{x^{\prime }}}.$$

For y∈Y, let $Q_{y}={\left(Q_{y|x}\right)}_{x}\in R^{X}$; then the utility of a mechanism Q:X→Y is given by $I_{P}\left(X;Y\right)=\sum_{y}\mu \left(Q_{y}\right)$. Furthermore, μ is a sublinear function in the sense of Definition 1 of [5]. We fix an ε>0. Furthermore, let $C\subset R_{\geq 0}^{X}$ be as in Lemma A8. Then, a mechanism Q satisfies $\left(\varepsilon ,P_{X}\right)$-RLDP if and only if each $Q_{y}$ is an element of C. Let V be the spanning set of V of Lemma A8, and let D be the polytope spanned by V. If Q satisfies ε-SLDP, then every column $Q_{y}$ is of the form $\theta_{y}\cdot d_{y}$, where $d_{y}\in D$ and $\theta_{y}\in R_{\geq 0}$ are such that $\sum_{y}\theta_{y}d_{y}=1_{X}$. Analogously to the proof of Theorems 2 and 4 in Section 7 of [5] (or, for that matter, our proof of Theorem 3), one proves that the optimal Q is found by taking b=a, and taking $d_{y}\in V$ for all *d*. Since

(A114) $$I\left(X;Y\right)=\sum_{y}\mu \left(Q_{y}\right)=\sum_{y}\theta_{y}\mu \left(d_{y}\right)$$

we can find the optimal Q by solving the following optimization problem, where $m\in R^{V}$ is the vector ${\left(\mu \left(v\right)\right)}_{v\in V}$, and where $A\in R^{X\times V}$ is the matrix whose *v*-th column is *v*:

$$\begin{array}{cc} {\mathrm{maximize}}_{\theta \in R^{V}}\; & m\cdot \theta \\ \mathrm{such}\mathrm{that}\; & A\cdot \theta =1_{X},\\ \; & \theta \geq 0. \end{array}$$

From here, we follow Section 9.5 of [5]. The dual to the above problem is

$$\begin{array}{cc} {\mathrm{minimize}}_{\alpha \in R^{X}}\; & (1_{X})\cdot \alpha \\ \mathrm{such}\mathrm{that}\; & A^{T}\cdot \alpha \geq m,\\ \; & \alpha \geq 0. \end{array}$$

By duality, we have $\max_{\theta }m\cdot \theta =\min_{\alpha }\left(1_{X}\right)\cdot \alpha$. We describe $\alpha^{*}\geq 0$ and $\theta^{*}\geq 0$, depending on ε, such that for sufficiently large ε one has $A^{T}\cdot \alpha^{*}\geq m$, such that $m\cdot \theta^{*}=\left(1_{X}\right)\cdot \alpha^{*}$ and $A\theta^{*}=1_{X}$, and such that $\theta^{*}$ corresponds to SRR, i.e., for each y∈Y=X there is a ${\hat{v}}_{y}\in V$ such that ${\mathrm{SRR}}_{y}^{\varepsilon }=\theta_{{\hat{v}}_{y}}^{*}{\hat{v}}_{y}$. Together, this proves that SRR is optimal for ε≫0. More concretely, for y=(s,u)∈X, define ${\hat{v}}_{y}$ by

(A115) $${\left({\hat{v}}_{y}\right)}_{s^{\prime },u^{\prime }}=\left\{\begin{array}{cc} e^{\varepsilon }, & \;\mathrm{if}\;\left(s^{\prime },u^{\prime }\right)=\left(s,u\right),\\ e^{-\varepsilon }, & \;\mathrm{if}\;s^{\prime }=s\mathrm{and}u^{\prime }\neq u,\\ 1, & \;\mathrm{if}\;s^{\prime }\neq s. \end{array}\right.$$

Note that ${\hat{v}}_{y}\in V$. Furthermore, let $\theta^{*}\in R^{V}$ be given by

(A116) $$\theta_{v}^{*}=\left\{\begin{array}{cc} \frac{1}{e^{\varepsilon }+e^{-\varepsilon }\left(a_{2}-1\right)+a-a_{2}}, & \;\mathrm{if}\;\mathrm{there}\;\mathrm{is}\;a\;y\in X\;\mathrm{such}\mathrm{that}v={\hat{v}}_{y},\\ 0, & \;\mathrm{otherwise}; \end{array}\right.$$

Then, SRR satisfies ${\mathrm{SRR}}_{y}^{\varepsilon }=\theta_{{\hat{v}}_{y}}^{*}{\hat{v}}_{y}$ for all y∈X, and for each x∈X one has

(A117)(Aθ*)x=∑vAx,vθv*(A118)=∑vvxθv*(A119)=∑y(v^y)xeε+e−ε(a2−1)+a−a2(A120)=1,

which shows that $A\theta^{*}=1_{X}$. Furthermore, define $\alpha^{*}\in R^{X}$ by

(A121) $$\alpha_{s,u}^{*}=c_{1}\mu \left({\hat{v}}_{s,u}\right)+c_{2}\sum_{u^{\prime }\neq u}\mu \left({\hat{v}}_{s,u^{\prime }}\right)+c_{3}\sum_{\begin{array}{c} s^{\prime }\neq s,\\ u^{\prime } \end{array}}\mu \left({\hat{v}}_{s^{\prime },u^{\prime }}\right),$$

where

(A122)c1=−(a2−2)(a2−1)+(a−a2+1)(a2−2)eε+(a−2a2+1)e2ε+e3ε(eε−1)(eε+1)(eε−a2+1)(eε+(a2−1)e−ε+a−a2),(A123)c2=a2−1+(a−a2+1)eε(eε−1)(eε+1)(eε−a2+1)(eε+(a2−1)e−ε+a−a2),(A124)c3=−e2ε(eε−1)(eε−a2+1)(eε+(a2−1)e−ε+a−a2).

A cumbersome but straightforward calculation shows that for all *x*, we have

(A125)m·θ*=(1X)·α*=1eε+e−ε(a2−1)+a−a2∑xμ(v^x),(A126)v^x·α*=mv^x=μ(v^x).

Furthermore, $c_{1},c_{2},c_{3}\geq 0$, so $\alpha^{*}\geq 0$. It remains to be shown that $\alpha^{*}$ satisfies the dual problem for ε≫0, i.e., $A^{T}\alpha \geq m$ for sufficiently large ε. To this end, for v∈V, set

(A127) $$\begin{array}{cc} F_{v} & =\{x\in X:v_{x}=e^{\varepsilon }\}, \end{array}$$

(A128) $$\begin{array}{cc} G_{v} & =\{x\in X:v_{x}=1\}, \end{array}$$

(A129) $$\begin{array}{cc} H_{v} & =\{x\in X:v_{x}=e^{-\varepsilon }\}, \end{array}$$

From the description of V in Lemma A8, we find that $|F_{v}|\geq 1$ for all *v*, and $|F_{v}|=1$ if and only if there exist s,u such that $v={\hat{v}}_{s,u}$. Now, write $P_{F_{v}}=\sum_{x\in F_{v}}P_{x}$ and likewise for $G_{v}$, $H_{v}$. For large ε, we have

(A130)mv=μ(v)=eε∑x∈FvPxlog1PFv+e−εPGv+e−2εPHv(A131)+∑x∈GvPxlog1eεPFv+PGv+e−εPHv(A132)+e−ε∑x∈HxPxlog1e2εPFv+eεPGv+PHv(A133)=−PFvlogPFveε+O(ε)

and furthermore

(A134)c1=e−ε+O(e−2ε),(A135)c2,c3=O(e−2ε).

From this, it follows that

(A136)αx*=c1μ(v^x)+(c2+c3)O(eε)(A137)=−PxlogPx+O(εe−ε),

Hence,

(A138) $$\begin{array}{cc} v^{T}\alpha^{*} & =\left(-\sum_{x\in F_{v}}P_{x}\log P_{x}\right)e^{\varepsilon }+O\left(\varepsilon \right). \end{array}$$

For $|F_{v}|\geq 2$, one has $P_{F_{v}}\log P_{F_{v}}>\sum_{x\in F_{v}}P_{x}\log P_{x}$. This means that if *v* is not of the form ${\hat{v}}_{x}$, one has $v^{T}\alpha^{*}\geq m_{v}$ for sufficiently large ε. Together with (A126), this shows that $A^{T}\alpha^{*}\geq m$ for sufficiently large ε; this concludes the proof. □
